# Microscale Near‐Neutral Zinc–Air Battery on Interdigitated Electrode Chips for High Current Operation

**DOI:** 10.1002/smtd.202501562

**Published:** 2025-11-10

**Authors:** Subhra R. Pattanayak, Nibagani Naresh, Yujia Fan, Yijia Zhu, Tharangattu N. Narayanan, Buddha Deka Boruah

**Affiliations:** ^1^ Tata Institute of Fundamental Research Hyderabad Serilingampally Mandal Hyderabad 500046 India; ^2^ Institute for Materials Discovery University College London London WC1E 7JE UK

**Keywords:** bifunctional cathode, high‐rate performance, micro zinc–air batteries, near‐neutral electrolyte, on‐chip energy storage

## Abstract

Microbatteries are essential for powering compact electronic devices, where traditional batteries fall short due to their bulk and limited integration flexibility. Recent progress in advanced materials, 2D/3D microfabrication techniques, and printable nonlithium chemistries has enabled significant improvements in performance, safety, and scalability. Among emerging technologies, microscale zinc–air batteries (ZAMBs) offer promise, delivering high energy density while utilizing safe, earth‐abundant materials and ambient oxygen as the cathodic reactant‐making them a lightweight, sustainable, and cost‐effective option for next‐generation miniaturized electronics. In this work, underexplored compact ZAMBs specifically designed for microdevices is presented. These are fabricated using directly electrodeposited catalysts and microplotted Pt/C as bifunctional cathodes, with zinc deposited on a porous silver scaffold of interdigitated electrodes serving as the anode. The resulting devices achieve an areal capacity exceeding 20 µAh cm^−2^ at a high areal current of 2 mA cm^−2^, along with a volumetric capacity of 85 mAh cm^−3^ sustained over 100 cycles‐outperforming previously reported most of microbatteries integrated on IDE platforms. Structural and electrochemical characterization confirmed the in situ formation of CoNi layered double hydroxide in the cathode, validating the feasibility of microscale bifunctional catalyst integration. Additionally, the use of a near‐neutral electrolyte ensures robust performance under mild operating conditions, supporting seamless incorporation into compact energy storage systems.

## Graphical abstract

Demonstration of zinc‐air rechargeable microbattery operating at high currents in near neutral electrolytes, as a promising solution for next generation, safe powering of miniaturised electronic gadgets.

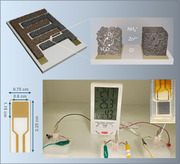

## 1 Introduction

The rapid miniaturization and functional expansion of electronic devices have created an urgent need for compact, safer, and energy‐efficient power sources. This has catalyzed the development of miniaturized medical implants,^[^ ^1^ ^]^ microrobots,^[^ ^2^ ^]^ environmental micro sensors,^[^ ^3^ ^]^ mini satellites,^[^ ^4^ ^]^ industrial sensor networks,^[^ ^5^ ^]^ compact power sources for autonomous systems and Internet of things (IoT) devices.^[^ ^6^ ^]^ These applications demand compact, safe, and energy‐dense on‐chip power sources capable of long‐term operation.^[^ ^7^ ^]^ While lithium‐ion micro‐batteries have been widely studied,^[^ ^8^ ^]^ they face safety risks, high fabrication costs, poor biocompatibility, and performance limitations in miniaturized systems.^[^ ^9^ ^]^ Zinc‐based micro‐batteries and micro‐capacitors are gaining attention for their intrinsic safety, low cost, and sustainability.^[^ ^10^, ^11^ ^]^ Among them, micro‐scale zinc‐air batteries (ZAMBs) stand out by offering higher energy and power densities compared to zinc‐ion micro‐batteries (ZIMBs) and micro‐capacitors,^[^ ^2^, ^12^, ^13^, ^14^ ^]^ making them promising candidates for advanced microsystems. ZAMBs using abundant materials and aqueous electrolytes further enhance safety, sustainability, and cost effectiveness. However, miniaturization of zinc‐air batteries (ZABs) is impeded by several challenges, including difficulties in achieving lithography‐compatible microfabrication,^[^ ^15^ ^]^ sluggish oxygen redox kinetics,^[^ ^16^ ^]^ and dendritic zinc growth.^[^ ^17^ ^]^ The present work addresses these lacunas by planar interdigitated electrode (IDE) based micro‐batteries using micro imprint processing.

Planar on‐chip architectures, enabled by micro imprint processing, minimize ion transport distance, increase structural robustness, and eliminate separators, offering superior energy efficiency and durability compared to conventional stacked designs, facilitating scalability for mass production. However, Schmidt et al. have tried to fabricate miniaturised ZAB mostly in stack designs using photolithography creating multiple domains in mm^2^ area.^[^ ^18^ ^]^ While Guo et al. have explored miniaturized ZAMBs using 3D‐printed IDE chips, their configuration with 1.2 mm finger width and 0.8 mm inter‐finger spacing remains outside the micro‐scale domain.^[^ ^19^ ^]^ Additionally, their performance relies on multilayer stacking, in contrast to our single‐layer micro‐battery performance study which has microscale dimensions. So far, no studies have focused on making miniaturized ZABs with micrometer‐scale widths (ZAMBs) using IDEs or on understanding their electrochemical behavior in detail. In this work, the ZAMBs were fabricated on Au IDEs ceramic substrates with a finger width of 200 µm and gap between two fingers being 50 µm. The fabrication involves using bifunctional cathode materials aiding for both the oxygen reduction reaction (ORR) and oxygen evolution reaction (OER), achieved through micro‐plotting of 20 wt.% Pt/C in one set. In another set, the electrodeposition time for Co–Ni layered double hydroxide (LDH) catalysts (CN) on Ag‐coated IDE chips was optimized to that miniaturized scale. The anodic electrode was prepared by depositing zinc onto a porous silver framework, providing improved adhesion^[^ ^20^ ^]^ and increased surface area. Although alkaline electrolyte is commonly used for ZAB for better activity, the near‐neutral gel electrolyte is considered here to avoid the harsh condition^[^ ^21^ ^]^ and study the cell performance. The synthesized ZAMBs demonstrate stable performance over 100 cycles at a significantly high areal current of 2000 µA cm^−2^. An areal capacity exceeding 20 µAh cm^−2^ is achieved at this areal current using conventional bifunctional catalysts such as Pt/C and electrodeposited CN materials. The volumetric capacity of >85 mAh cm^−3^ is observed till 100 cycles for Pt‐ZAMBs. This shows a tremendous opportunity in the fabrication of ZAMBs for the next‐generation miniaturized electronics.

## 2 Results and Discussion

**Figure 1:**
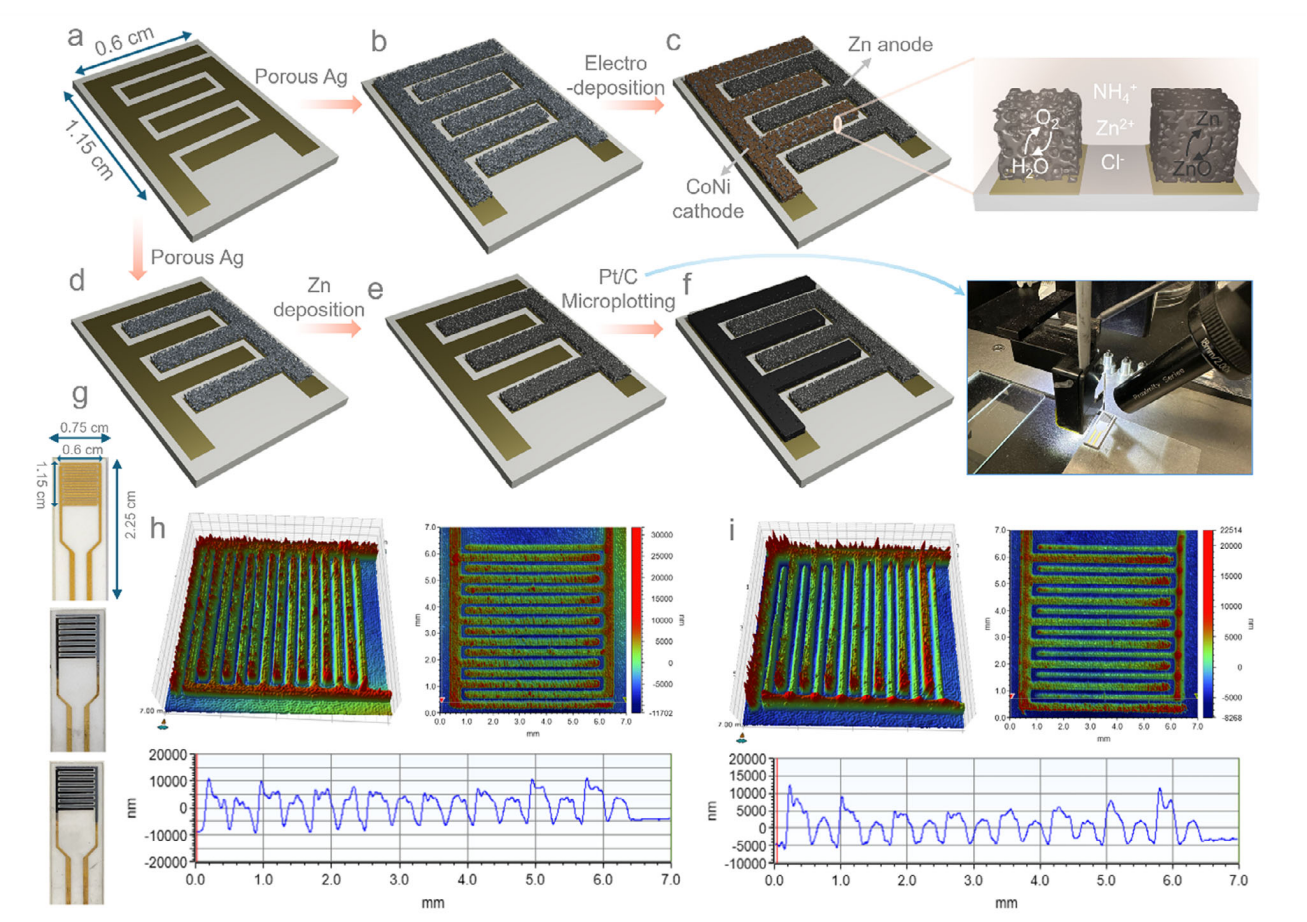
Schematic representation of the fabrication steps of ZAMBs: a) Au IDE having interdigitated area (1.15 cm × 0.6 cm) b) porous Ag scaffold electrodeposition on IDE. c) Zn and Co/Ni electrodeposition for anode and cathode respectively to fabricate CN‐ZAMB (Inset of the figure illustrates the reversible Zn ⇌ ZnO conversion at the anode and the bifunctional ORR/OER processes at the cathode, facilitated by the presence of electrolyte ions such as NH_4_⁺, Zn^2^⁺, and Cl^−^ and H_2_O molecules). d) Ag electrodeposition on right side of Au IDE chip and e) Zn electrodeposition on the top of porous Ag. f) Pt/C microplotting on the left side of Au IDE to fabricate Pt‐ZAMB. g) Digital photographs of the Au IDE chip having total area 2.25 cm × 0.75 cm) and active area (1.15 cm × 0.6 cm), CN‐ZAMB and Pt‐ZAMB. h,i) 2D and 3D profilometer images of the CN‐ZAMB and Pt‐ZAMB, respectively, with the lower images displaying electrode height profiles: both configurations show a Zn anode height of ≈7 µm. The CN cathode in CN‐ZAMB measures ≈5 µm in height, while the Pt/C cathode in Pt‐ZAMB is ≈2 µm. In both cases, these measurements exclude the flat Au IDEs thickness of ≈4 µm.

To fabricate the ZAMBs, zinc (Zn) was used as the anode material, and it was electrodeposited on the porous silver framework that is patterned onto the flat IDEs of gold (Au) using dynamic hydrogen bubble template (DHBT) electrodeposition (the details of the parameters are mentioned in the experimental section). In this technique, high applied potential forms gas bubbles at the electrode surface, acting as soft templates to create porous or structured Ag coatings.^[^ ^22^ ^]^ Optimization in the deposition time leads to structured morphological porosity features (Figure S1, Supporting Information). As Zn was leaching out completely from the micro electrode by just merely dipping in the aqueous solvent for 1 h, the stability of the Zn on the Au surface of the IDE was enhanced by introducing a porous silver interlayer prior to the Zn deposition (Figure S2, Supporting Information) as seen from the data. **Figure** **1**a shows Au chip micro electrode. To fabricate CN‐ZAMB, i.e., a full cell having Zn electrodeposited on one side and CN electrodeposited on another side of the chip, Figure 1b is the first step showing Ag electrodeposition on both sides of the interdigitated electrodes. Figure 1c illustrates the formation of the CN‐ZAMB (details of the procedure are mentioned in Experimental Section).

The bifunctional cathode catalyst was electrodeposited using the electrodeposition method^[^ ^23^ ^]^ and the deposition time period was optimized for micro fingers of the IDE chips. The synthesized compounds on the cathode side are characterized to be CN; details of the characterization are discussed later. To fabricate Pt‐ZAMB (Figure 1d), Ag was electrodeposited on one side of the chip, followed by Zn electrodeposition on the Ag layer (Figure 1e), using the same deposition time as that used for CN‐ZAMB. However, for the cathode development of Pt‐ZAMB, instead of electrodeposition, 20 wt.% Pt/C is microplotted (details of the microplotted deposition procedure are mentioned in the literature).^[^ ^24^ ^]^ As 20 wt.% Pt/C is considered the benchmark cathode catalyst for ZABs,^[^ ^25^, ^26^, ^27^ ^]^ hence considered to microplot (Figure 1f) as cathode material.

Neutral chloride‐based electrolytes are preferred over alkaline ones in rechargeable ZABs because they suppress Zn corrosion/dendrite growth, prevent carbonate formation, and extend cycling stability. They also enable stable quasi‐solid/flexible designs with better hydrogel durability, as alkaline gels suffer structural degradation under high pH. Initial research on neutral rechargeable ZABs identified chloride‐based inorganic salt solutions as strong electrolyte candidates.^[^ ^28^ ^]^ In ZnCl_2_–NH_4_Cl electrolytes, ZnCl_2_ supplies Zn^2+^ ions for smooth deposition/dissolution at the Zn anode, while NH_4_Cl buffers the pH to minimize corrosion.^[^ ^29^ ^]^ This synergy enhances cycling stability and durability, making the system well‐suited for small‐scale ZABs employing Pt/C as the cathode catalyst.^[^ ^30^ ^]^ The electrolyte was composed of optimized amount of 2.34  M NH_4_Cl, 0.51 m ZnCl_2_, 1000 ppm thiourea, and 1000 ppm polyethylene glycol (PEG).^[^ ^31^ ^]^ To get a gel‐like consistency to the electrolyte, which helps to reduce evaporation, 1 g of polyvinyl alcohol (PVA) was added to 10 mL of the electrolyte and heated at 80 °C for 2 h. The slurry was cooled to obtain a neutral gel electrolyte. The inset of Figure 1f illustrates the redox processes occurring at each electrode during cycling.

During the discharge, oxygen (dissolved oxygen) is reduced to water at the cathode, while the anode undergoes Zn to ZnO conversion. Upon charging, corresponding reverse reactions take place at both electrodes.^[^ ^31^ ^]^ Figure 1g shows digital images of the devices, such as Au chip as Au‐IDE, CN‐ZAMB, and Pt‐ZAMB. To understand the thickness of the microelectrodes in the micro‐batteries, profilometer scanning was performed. Figure 1h,i demonstrates 2D and 3D views with the height profiles of CN‐ZAMB and Pt‐ZAMB respectively, confirming successful microbattery fabrication without short circuits. The measured average thicknesses of the Zn anode and CN cathode are 7 and 5 µm, respectively (considering flat Au IDEs, which are ≈4 µm^[^ ^32^ ^]^). The average thickness of the Zn anode and Pt/C microplotted cathode is 7 and 2 µm, respectively. The average thickness of the microplotted cathode is more than 2 times less than the electrodeposited cathode. This shows that such a controlled thickness coating can be obtained by the microplotting method.

**Figure 2:**
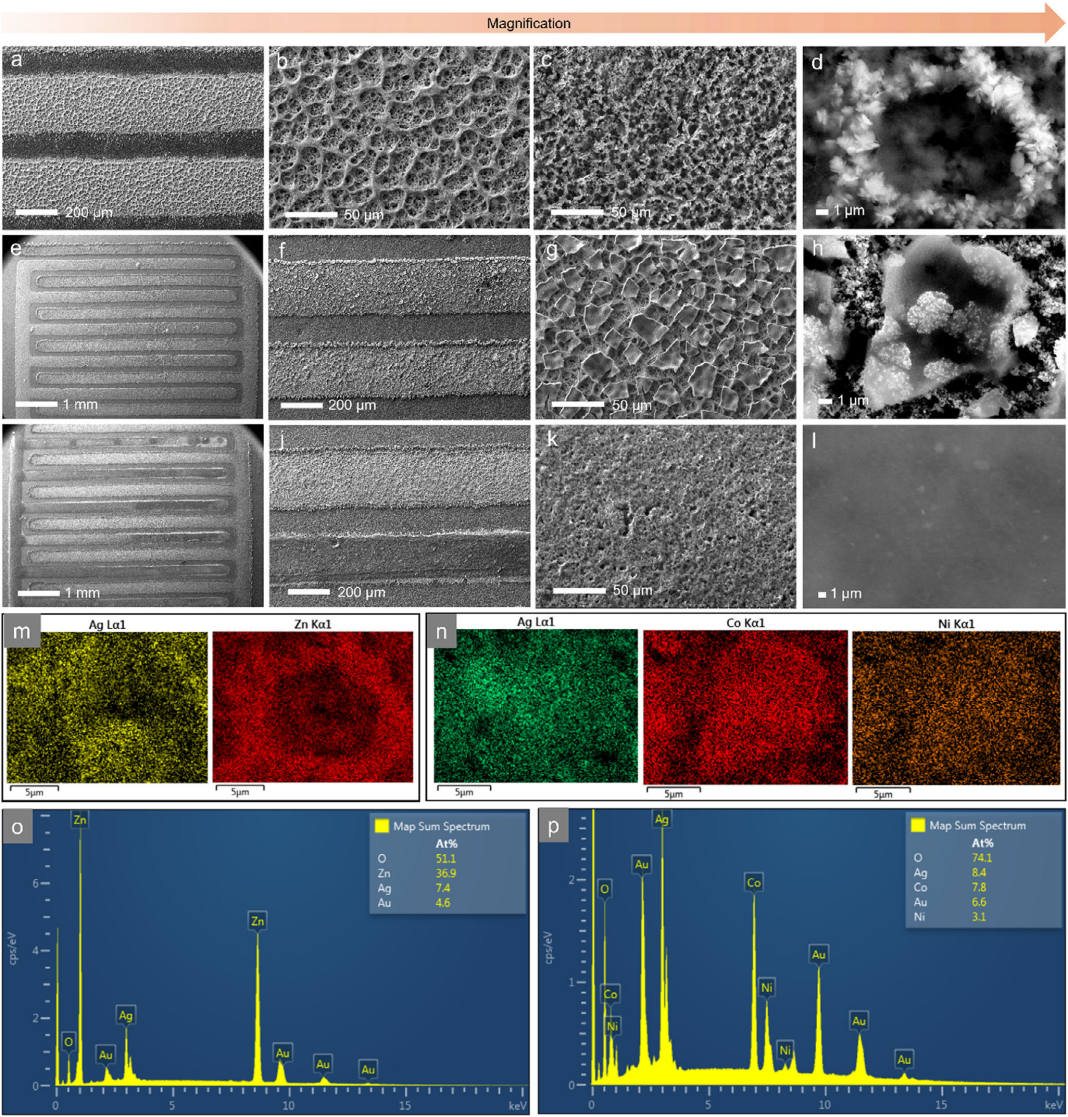
SEM images at different magnifications and EDS characterization of the samples. a,b) SEM images of porous Ag. c,d) SEM images of electrodeposited Zn on porous Ag. e) SEM image of CN‐ZAMB full cell. f) Selective Zn and CN electrodeposition on two adjacent IDE fingers, forming the anode and cathode, respectively. g,h) Different magnification of CN cathode. i) SEM image of Pt‐ZAMB full cell. j) Selective Zn and Pt electrodeposition on two adjacent IDE fingers, forming the anode and cathode, respectively. k,l) Different magnification of Pt/C cathode. EDS mapping showing presence of m) Ag, Zn on anodic side and n) Ag, Co, Ni on cathodic side of CN‐ZAMB. Elemental analysis by EDS showing atomic percentage of o) Ag, Zn, O in anode and p) Ag, Co, Ni, O in CN cathode.

The as synthesized electrodes are characterized by Scanning electron microscopy (SEM) technique. **Figure** **2**a,b demonstrates porous morphology of Ag on Au chip at different magnifications (average thickness of the Ag layer is observed to be 3.5 µm from the profilometry data, Figure S3, Supporting Information). Figure 2c,d shows Zn electrodeposition on the as‐deposited porous silver at different magnifications. The Zn is electrodeposited on the edge of the pores of the Ag; the pore sizes are distributed up to ≈15 µm (Figure S4, Supporting Information) which is advantageous for effective electrolyte interaction on the anodic surface, potentially helpful for redox process. The Figure 2e,f demonstrate successful deposition of the anodic and cathodic materials of CN‐ZAMB precisely without any short circuit problem under optimal deposition condition. Figure 2g,h show as deposited CN cathode materials at different magnifications indicating growth of the micrometre size flakish metallic deposition on the Ag scaffold. The Ag scaffold provides better adherence to the corresponding anodic and cathodic materials (discussed in detail later). Figure 2i,j show precise microplotting of the Pt/C cathodic material next to the anodic deposition avoiding short circuiting. This shows significance of microplotting method as a physical deposition process and controlled electrodeposition as a technique to fabricate microlevel devices. Figure 2k shows Au surface after microplotting indicating very thin deposition as surface morphology shows some structural feature of plane Au electrode (Figure S5, Supporting Information). Figure 2l is the magnified view of the corresponding Pt/C cathode that shows the absence of any characteristic morphology as it's the slurry coating on the Au electrode. Figure 2m is the corresponding energy dispersive spectroscopy (EDS) mapping of the Figure 2d, which confirms presence of Ag and Zn in as demonstrated porous morphology with Zn spectra coming more prominent on the porous Ag edges. The Figure 2n shows corresponding EDS mapping of the Figure 2h confirming the presence of Ag, Co, and Ni in the cathode material. Co and Ni spectra coming more prominently from the as‐observed flakish deposition confirms that the flakes are due to CN deposition. The Figure 2o shows elemental analysis of anodic side (Figure 2m) showing the atomic percentages (%) as ≈37% Zn, ≈51% O, and ≈7% Ag. Moreover, this is indicating that surface coverage is mainly dominated by Zn, and high oxygen atomic % indicates presence of absorbed oxygen or from some surface oxidized Zn. The Figure 2p shows elemental analysis of cathodic side (Figure 2n) showing atomic % of ≈8% Co, ≈3% Ni, ≈8% Ag, and ≈74% O indicating surface of the cathode contains Co/Ni at an atomic ratio of Co to Ni more than 2 times. Point EDS (Figures S6–S8, Supporting Information) confirms uniform Co/Ni distribution across both flake‐like and nonflake‐like regions. The absorbed oxygen or hydroxide species contribute a very large atomic percentage as this does not solely come from lattice oxygen.

**Figure 3:**
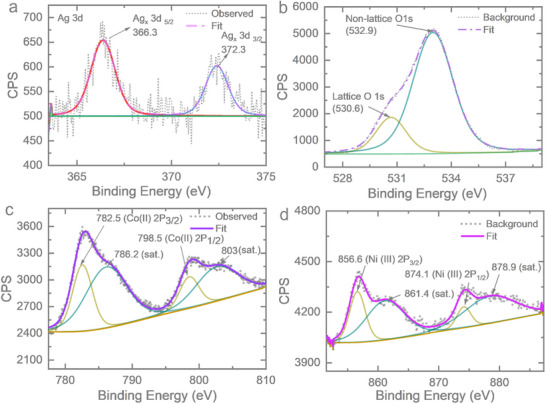
Surface characterization of electrodeposited CN. XPS high resolution spectra for a) Ag 3d_5/2_, 3d_3/2_ b) O 1s c) Co (II) 2p_3/2,_ 2p_1/2_ d) Ni (III) 2p_3/2,_ 2p_1/2_.

To gain deeper insight into the chemical composition of the electrodeposited cathodic sample, high‐resolution X‐ray photoelectron spectroscopy (XPS) was performed on samples deposited onto Cu foil. The spectra were evaluated in CasaXPS and the Tougaard background was selected due to its reduced sensitivity to the choice of minimum and maximum energy limits, making it more reliable than the Shirley background, especially when fitting over large energy ranges.^[^ ^33^ ^]^ The high resolution XPS spectra of Ag shows peak at 366.3 eV for 3d_5/2_ and 372.3 eV for 3d_3/2_. This ≈6 eV difference corresponds to spin‐orbit coupling for silver.^[^ ^34^ ^]^ The pronounced shift (≈1.9 eV) to lower binding energy relative to metallic Ag, and ≈1.3 eV compared to AgO, suggests strong electronic interactions with the transition metals. In contrast, without Co or Ni deposition, the binding energy of Ag indicates the presence of metallic Ag and AgO (Figures S10, Supporting Information). **Figure** **3**b shows peaks at 530.6 and 532.9 eV for 1s of oxygen atom (O), which can be attributed to surface lattice oxygen from metal‐oxygen bond^[^ ^35^ ^]^ and adsorbed water, respectively.^[^ ^36^ ^]^ Although XPS is widely used to study the surface chemistry and electronic structure of nickel (Ni) and cobalt (Co) compounds, data interpretation remains complex. This complexity arises from 3d orbital multiplet splitting in tetrahedral or octahedral coordination environments, coupled with characteristic satellite structures at higher binding energies caused by multielectron excitations (e.g., shake‐up processes).^[^ ^37^ ^]^ The high‐resolution XPS spectra of the Co exhibits a characteristic spin‐orbit doublet involving 2p orbitals with a binding energy separation of 16 eV between the Co 2p_3/2_ at 782.5 eV and Co 2p_1/2_ peak at 798.5 eV, indicating the presence of Co^2+^ species.^[^ ^33^ ^]^ The broad satellite peak at ≈786.2 and 803 eV indicates the presence of oxide species of Co.^[^ ^38^ ^]^ The high‐resolution XPS spectra for Ni 2p illustrate the Ni (III) 2p_3/2_ at 856.6 eV and Ni(III) 2p_1/2_ peak at 874.1 eV, having shake‐up satellite peaks at binding energies of 861.4 and 878.9 eV, respectively.^[^ ^23^ ^]^ The survey spectrum is provided in Figure S9 (Supporting Information). The XPS spectrum of Zn deposited on Ag (Figure S11, Supporting Information) reveals that, unlike the electronic interactions between Ag and transition metals on the cathodic side, the anodic Ag exists as a metallic state with surface oxidation to Ag(I). The presence of metallic Zn further confirms that Zn deposited on Ag is not leading to an alloy formation. Then Raman spectroscopy was carried out with 532 nm laser (details are in the Experimental Section). Bulk Ag is not Raman active in the 200–1000 cm^−1^ region because it has a highly symmetric face‐centered cubic (fcc) structure with no first‐order Raman active modes. However, electrodeposition of Ag induces surface enhanced Raman scattering (SERS), which increases the intensity of the otherwise feebly observed CuO peaks at 526 and 614 cm^−1^.^[^ ^39^, ^40^ ^]^ The Raman analysis after electrodeposition indicates that it exhibits superposition of various Raman peaks with new peaks generated at 462 and 531 cm^−1^ which is attributed to Ni‐OH/Co‐OH and Ni‐O/Co‐O stretching modes of Co‐Ni LDH^[^ ^41^, ^42^, ^43^ ^]^ (Figure S12, Supporting Information).

The X‐ray diffraction spectroscopy (XRD) was carried out to obtain as as‐deposited crystal structure. In the obtained spectra (Figure S13, Supporting Information), three prominent peaks at 43.3°, 50.4°, and 74.2° correspond to the (111), (200), and (220) planes of the face‐centered cubic (fcc) structure of Cu, as identified from ICDD file No. 70‐3039, respectively.^[^ ^44^ ^]^ Characteristic peaks at 2θ values of 38.1°, 44.3°, 64.5°, and 77.5° correspond to the (111), (200), (220), and (311) planes of face‐centered cubic (fcc) Ag, which are in agreement with the standard ICDD file No. 89–3722.^[^ ^45^ ^]^ The peak at 10.9° is indicative of the (003) basal reflection of the layered double hydroxide (LDH) structure, confirming the presence of the Co–Ni LDH phase (ICDD file No. 22‐0444) in the sample.^[^ ^44^, ^46^ ^]^

### 2.1 Micro‐Battery Performance

After the fabrication of the electrodes, the cell (battery) performance was studied. The stability of the Zn on the Au electrode was tested in the neutral gel electrolyte consisting of 2.34 m NH_4_Cl, 0.51 m ZnCl_2_, 1000 ppm ZnO, and 1000 ppm PEG. The as‐prepared Ag/Zn//Ag/Zn symmetric cell showed 53% better capacity in the first cycle and 23% better capacity in the 10th cycle than the Zn//Zn symmetric cell (Figure S14, Supporting Information). Precise mass measurements on IDEs are difficult due to their limited active area (≈0.2 cm^2^); thus, mass loading is not provided. However, the SEM data show uniform distribution of the materials on the anodic and cathodic sides (Figure 2).

**Figure 4:**
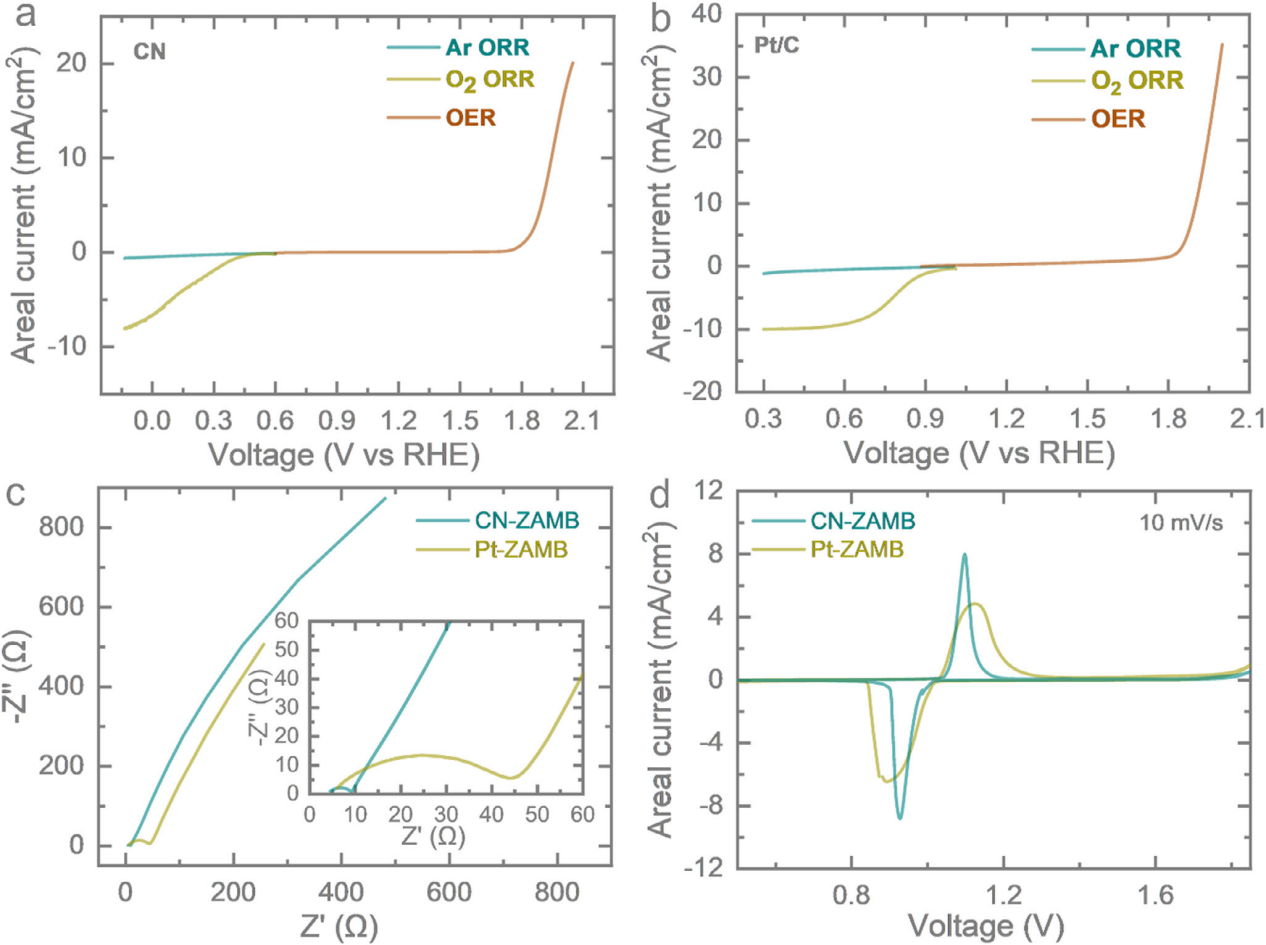
Half‐cell ORR and OER performance test for a) CN cathode and b) Pt/C cathode. c) Ion transfer resistance study by EIS, d) CV at 10 mV S^−1^ scan rate, showing a sharp peak for battery‐like energy storage.

To know the bifunctional catalytic behavior of the CN and Pt/C catalysts, half‐cell measurements with them as working electrodes were carried out in a 3‐electrode setup (details are in the experimental section). **Figure** **4**a shows that in the absence of dissolved O_2_ in electrolyte (ensured by Ar purging), no Faradaic reduction current is observed, whereas after O_2_ saturation, the CN electrode exhibits ORR activity with an onset potential of 0.38 V vs RHE. Additionally, the OER activity is observed with an onset potential of 1.83 V vs RHE, confirming the bifunctional behavior of the CN catalyst. The ORR and OER onset potentials for Pt/C are found to be at 0.92 V vs RHE and 1.81 V vs RHE, respectively (Figure 4b). This confirms the system operates as an air battery, utilizing O_2_ as the active cathode material. To further establish the catalytic activity of the developed CN and Pt/C electrodes, electrochemical impedance spectroscopy (EIS) was performed at open circuit potential (OCP) conditions using the fabricated microelectrodes (details of the conditions are mentioned in the experimental section). The results show that the electrodeposited sample exhibits lower ion diffusion resistance compared to the microplotted Pt/C sample (Figure 4c). This is because direct electrodeposition creates a stronger electrode–catalyst interface compared to physical deposition methods that use binder materials. Cyclic voltammetry (CV) of the full cell shows a distinct diffusion‐limited peak (Figure 4d), indicating battery‐type energy storage behavior of the as‐synthesized electrode. Furthermore, the electrochemical performance of the fabricated ZAMB was systematically investigated. The overall reaction on the two electrodes is provided by the following Equations (1) and (2).^[^ ^31^ ^]^

Overall Reactions:

Anode–

(1) $$\mathrm{Zn}+H_{2}O\underset{\mathrm{charge}}{\overset{\mathrm{discharge}}{⇄}}\;\mathrm{ZnO}+2H^{+}+2e^{-}$$

Cathode–

(2) $$O_{2}+4H^{+}+4e^{-}\underset{\mathrm{charge}}{\overset{\mathrm{discharge}}{⇄}}\;2H_{2}O$$

**Figure 5:**
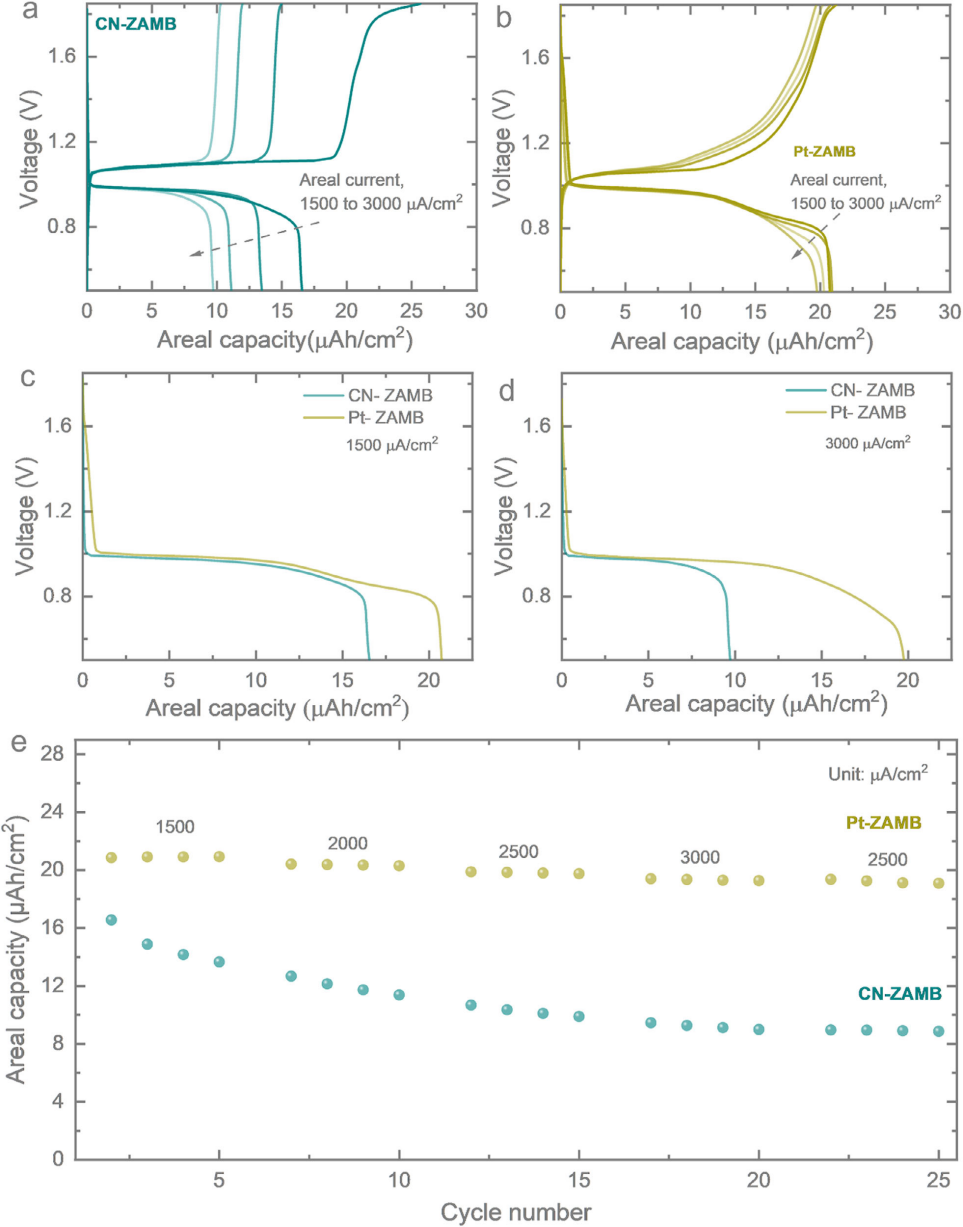
GCDs of a) CN‐ZAMB and b) Pt‐ZAMB at different areal currents ranging from 1500 to 3000 µA cm^−2^ over a voltage window of 0.5–1.85 V. c,d) Comparative GCDs of the CN‐ZAMB and Pt‐ZAMB show a significant increase in areal capacities from 16.6 to 20.7 and 9.6 to 19.8 µAh cm^−2^ at areal currents of 1500 and 3000 µA cm^−2^, respectively. e) Rate test of the CN‐ZAMB and Pt‐ZAMB at different areal currents, increasing from 1500 to 3000 µA cm^−2^ and then back to 2500 µA cm^−2^, showing corresponding areal capacity variation.

The Pt/C shows n ≈ 3.5 (majorly 4e^−^ ORR pathway), while n for CN catalyst could not be precisely determined due to electrodeposition on Au IDE (details are provided in Figures S15 and S16, Supporting Information). The open circuit voltage of the CN‐ZAMB and Pt‐ZAMB was measured, and they were found to be ≈0.95 V (Figure S17, Supporting Information). The galvanostatic charge–discharge (GCD) measurements were conducted for both CN‐ZAMB and Pt‐ZAMB across a range of very high areal currents, from 1500 up to 3000 µA cm^−2^. The corresponding GCD profiles were recorded within a voltage window of 0.5–1.85 V for CN‐ZAMB and Pt‐ZAMB, depicted in **Figure** **5**a,b, respectively. The Pt‐ZAMB exhibits a substantial improvement in areal capacity relative to the CN‐ZAMB in line with the half‐cell performance. At 1500 and 3000 µA cm^−2^, the Pt‐ZAMB delivers comparative areal capacities of 16.6–20.7 µAh cm^−^^2^ and 9.6–19.8 µAh cm^−2^, respectively nearly doubling the values obtained from the CN‐ZAMB at higher areal current. Rate performance comparisons between CN‐ZAMB and Pt‐ZAMB (Figure 5e) further validate the superior capacity retention and reversibility of the Pt‐ZAMB. At areal currents of 1500, 2000, 2500, 3000 µA cm^−2^, the Pt‐ZAMB achieves areal capacities of ≈21, 20.4, 19.9 and 19.3 µAh cm^−2^, respectively and again returning to 2500 µA cm^−2^, the areal capacity is obtained as 19.3 µAh cm^−2^ which highlights the Pt‐ZAMB's ability to sustain both high areal capacity and rate performance. The CN‐ZAMB though started from 16.4 µAh cm^−2^ at an areal current of 1500 µA cm^−2^ but reaches to 13.6 µAh cm^−2^ even in 4 cycles which indicates a possible leaching or change in chemical activity of the as‐deposited sample. However, after initial steep decrement, the slope decreased and in 2000, 2500, 3000 µA cm^−2^, the areal capacity changed from 12.7 to 11.3, 10.7 to 10, 9.5 to 9 µAh cm^−2^, in 4 cycles respectively. Again, returning to a real current of 2500 µA cm^−2^, the areal capacity remained ≈9 µAh cm^−2^ in all 4 cycles. This shows though a better interfacial connection is formed, still the intrinsic activity of the material and stability of the active material over the electrode surface determine the capacity performance and rate performance of the battery. The discharge polarization curves with the corresponding power density and energy density were evaluated for both Pt‐ZAMB and CN‐ZAMB (Figures S18 and S19, Supporting Information). Experimentally, the power density of CN‐ZAMB was higher than that of Pt‐ZAMB due to its higher discharge voltage at a given current density. However, Pt‐ZAMB sustained discharge for a longer duration at each current density, which results in a higher overall energy density compared to CN‐ZAMB. Thus, CN‐ZAMB exhibited superior discharge voltage characteristics, whereas Pt‐ZAMB delivered longer operational stability.

**Figure 6:**
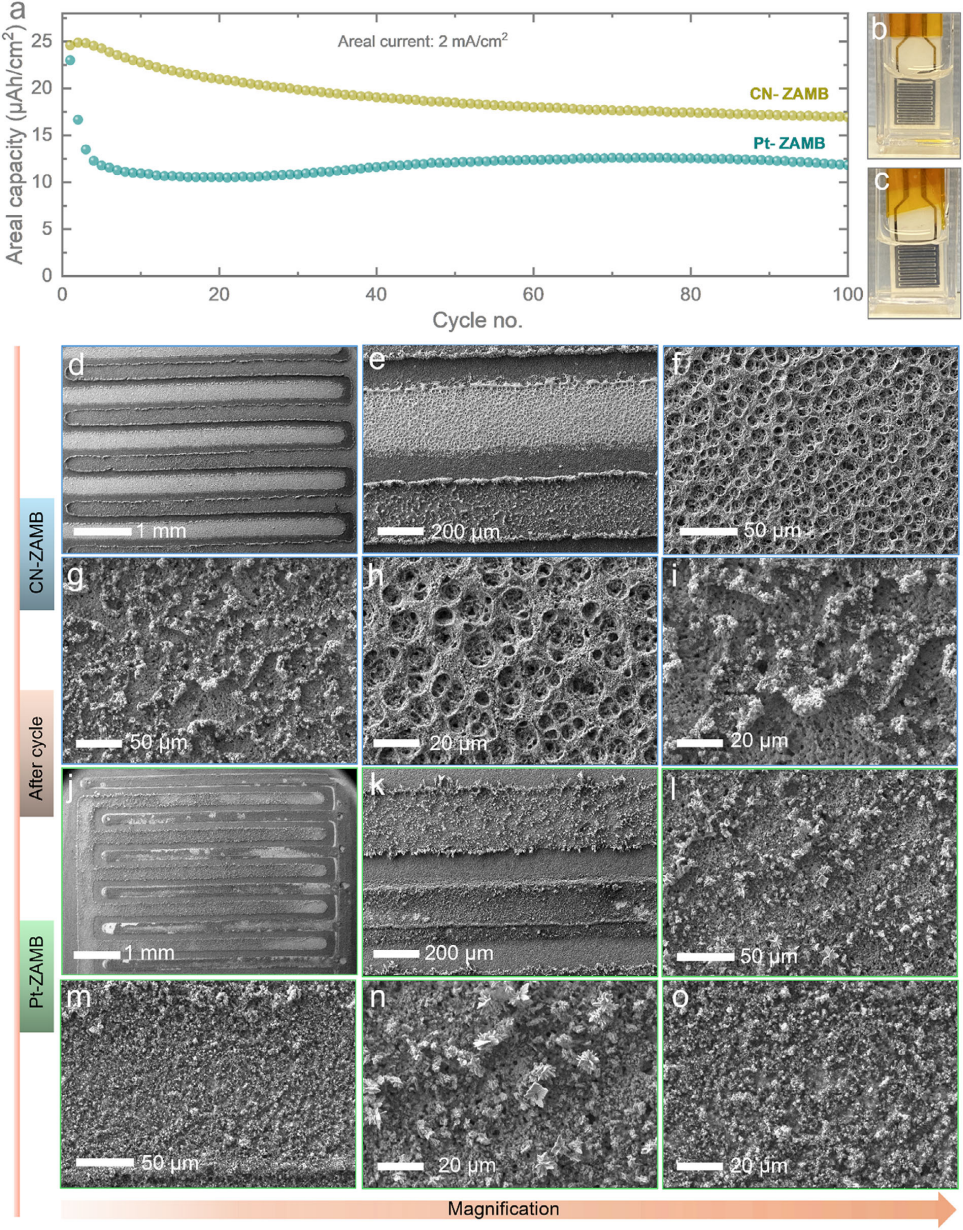
a) Extended long‐term cycling tests of the CN‐ZAMB and Pt‐ZAMB for 100 cycles, with observed capacity retentions of ≈50% and ≈76% for CN‐ZAMB and Pt‐ZAMB, respectively. Digital images of the cycled micro‐batteries b) CN‐ZAMB and c) Pt‐ZAMB d,e) Postmortem SEM images of a cycled CN‐ZAMB at different magnifications. f,h) High magnification images of the Zn anode side after cycling showing Zn loss from porous Ag, g,i) cathode degradation due to cycling j,k) SEM images of cycled 3D Pt‐ZAMB l,n) Anode shows presence of Zn with incipient dendritic structures m,o) presence of micro plotted Pt/C cathode material after cycling.

Subsequently, extended GCD cycling was carried out for both CN‐ZAMB and Pt‐ZAMB over 100 cycles in 2000 µA cm^−2^, as illustrated in **Figure** **6**a. The results demonstrate stable cycling behavior for both devices. The areal capacities of 23 and 25 µAh cm^−2^ were observed for CN and Pt/C based ZAMB systems, respectively. The GCD carried out for Pt/C at 3 mA cm^−2^ is shown in Figure S20 (Supporting Information) that shows areal capacity >40 µAh cm^−2^ for ≈20 cycles that indicates high areal capacity at higher current density is possible by stabilizing the anodic and cathodic processes happening in ZAMBs).

The capacity retentions were measured at 50% for CN‐ZAMB and 76% for Pt‐ZAMB after 100th cycle and the values stabilized at ≈18 and ≈12 µAh cm^−2^ for Pt‐ZAMB and CN‐ZAMB, respectively. The volumetric capacity remains >85 mAh cm^−3^ for Pt‐ZAMB and >25 mAh cm^−3^ for CN‐ZAMB (Figure S21, Supporting Information) for 100 cycles. Both the systems exhibit ≈100% coulombic efficiency (Figures S22 and S23, Supporting Information). Although CN‐ZAMB showed a 20% initial drop (Figure S22, Supporting Information), Pt‐ZAMB cell maintained 100% efficiency throughout (Figure S23, Supporting Information). These findings confirm that Pt‐ZAMB preserves its charge storage capability over 100 cycles, maintaining enhanced capacity values without having parasitic loss confirmed from coulombic efficiency (CE) data. The observed degradation beyond 100 cycles is primarily attributed to the loss of cathode and anode materials.

To further study the structural changes in the microelectrodes with cycling, ex situ SEM analysis was conducted. The digital photographic images of the post‐cycled CN‐ZAMB, Pt‐ZAMB devices are provided in Figure 6b,c, respectively. SEM analysis of the cycled CN‐ZAMB (Figure 6d,e) at low magnification confirmed the absence of short‐circuiting. Higher magnification images of the anode (Figure 6f,h) revealed that Zn has come out from the surface, as evidenced from the observed porous Ag morphology. Higher magnification images of the electrodeposited cathode (Figure 6g,i) also show a large amount of material loss including electrodeposited Ag layer (compared to the bare Au chip). This is likely due to stronger alloying, which causes the Ag to be stripped off along with the deposited Co/Ni during cycling. Hence, the areal capacity decrement to 50% in this sample can be majorly attributed to active material loss from the electrode. SEM analysis of the cycled Pt‐ZAMB (Figure 6j,k) at low magnification confirmed the absence of short‐circuiting. The edge of the Zn electrode shows dendritic growth, which is consistent with the elevated electric field concentrations at electrode edges and the lateral Zn^2+^ ion diffusion, contributing to dendrite formation, and high magnification images (Figure 6l,n) show the presence of some amount of Zn on the anodic fingers. The cathodic side (Figure 6m,o) also shows presence of Pt/C however the metallic shine is due to the loss of some amount of surface binder though further clarification regarding the chemical nature is difficult to obtain. This shows that material loss, inhomogeneous deposition, and modification of the chemical nature are major reasons for the observed capacity loss in both samples.

**Figure 7:**
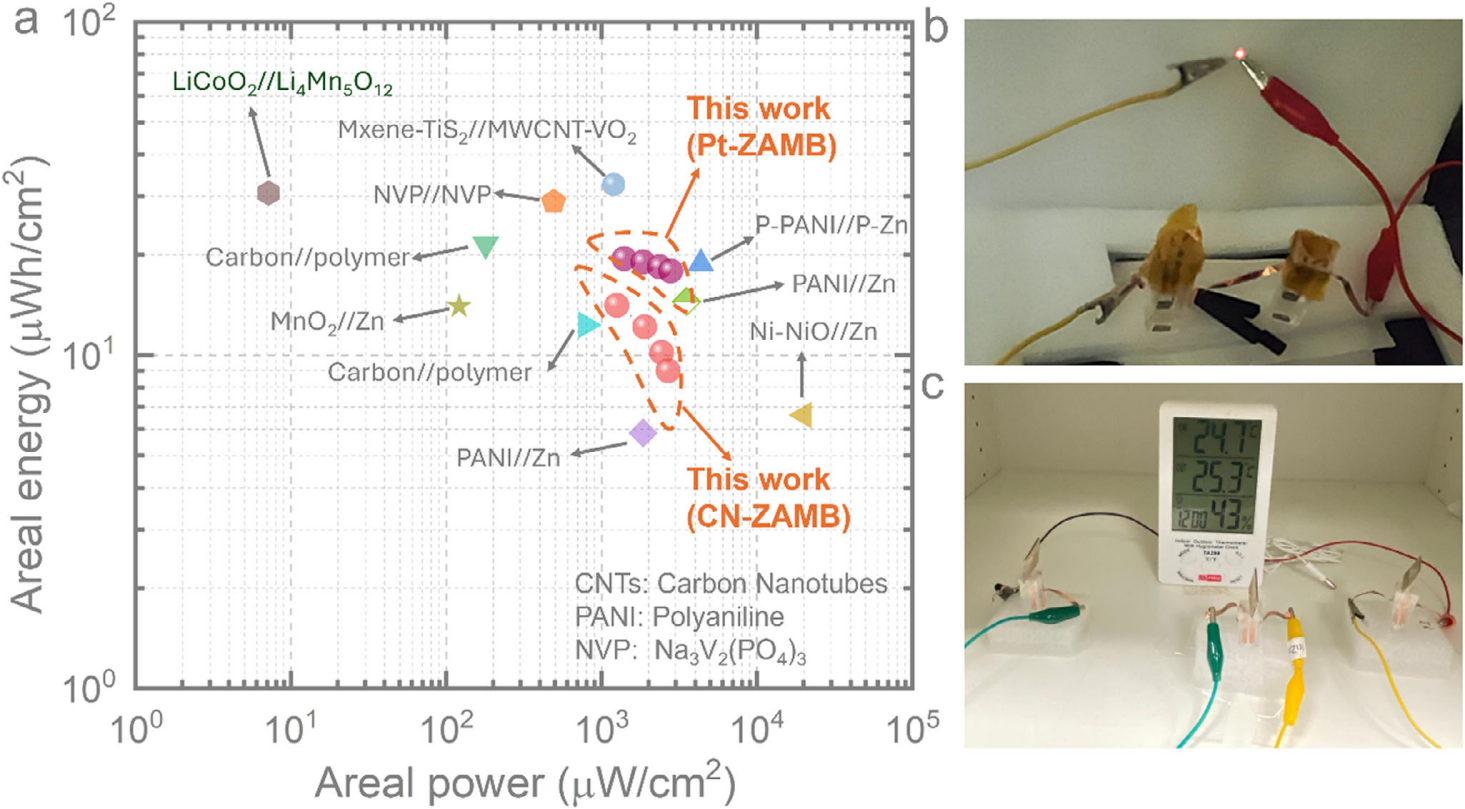
a) Ragone plot comparing the areal energy and areal power performance of the CN‐ZAMB and Pt‐ZAMB with previously reported various types of MBs: P‐PANI//P‐Zn,^[^ ^47^ ^]^ Carbon//Polymer,^[^ ^48^ ^]^ PANI//Zn,^[^ ^49^ ^]^ Ni‐NiO//Zn,^[^ ^50^ ^]^ Ni//Bi,^[^ ^51^ ^]^ LiCoO_2_//Li_4_Mn_5_O_12_,^[^ ^52^ ^]^ Zn//MnO_2_,^[^ ^53^ ^]^ NVP//NVP,^[^ ^54^ ^]^ Mxene‐TiS_2_//MWCNT‐VO_2_,^[^ ^55^ ^]^ Flat Au PANI//Zn,^[^ ^32^ ^]^ b) Two CN‐ZAMB cells in series to glow a 1.5 V LED, c) three Pt‐ZAMBs in series effectively powered an indoor–outdoor thermometer with a hygrometer clock.

We next compare the areal energy and power densities of our CN‐ZAMB and Pt‐ZAMB with those of previously reported micro‐batteries, as shown in the Ragone plot (**Figure** **7**). The Pt‐ZAMB achieves a maximum areal energy of 19.47 µWh cm^−2^ and a peak areal power of 2799 µW cm^−2^, outperforming most on‐chip micro‐batteries and thin‐film batteries reported to date. Notably, the Pt‐ZAMB not only delivers higher areal energy at a high current density of 2 mA cm^−2^ but also matches the areal power of leading micro‐supercapacitors. Meanwhile, the CN‐ZAMB demonstrates strong performance as well, with a maximum areal energy of 14.10 µWh cm^−2^ and an areal power of 2690 µW cm^−2^ (Figure 7). These findings highlight the efficacy of our advanced micro‐electrode architecture and innovative ZAMB design, which enable efficient energy storage even at high current densities within a compact device footprint. This work represents a significant step forward in the development of high‐performance on‐chip energy storage solutions, which are essential for next‐generation smart system‐on‐chip technologies.

To provide oxygen to the cathode, the upper section of the cell was left uncovered, allowing direct access to ambient air. Since the microscale on‐planar ZAB is investigated in this work, it is compared with such microscale on‐planar batteries in the Ragone plot given in Figure 7a consists of several microbatteries, including Zn and Li based micro ion batteries, sodium ion battery, Carbon‐microelectromechanical systems (C‐MEMS), nickel–bismuth (Ni//Bi) based planar microbatteries. To demonstrate the applicability of these fabricated microbatteries, we have connected two CN‐ZAMB cells in series that can glow a 1.5 V LED (Figure 7b) and three Pt‐ZAMBs in series, effectively powering an indoor‐outdoor thermometer with a hygrometer clock (Figure 7c), highlighting the potential for broader applications of on‐chip micro air batteries.

## 3 Conclusion

In summary, the ZAMB fabricated within a minimal footprint‐using directly electrodeposited CN catalysts and micro‐plotting Pt/C on IDEs‐demonstrates strong bifunctional oxygen electrocatalytic activity, as evidenced by both half‐cell and full‐cell performance. The devices deliver an areal capacity exceeding 20 µAh cm^−2^, making it competitive among ZIMBs with similar IDE‐patterned architectures. Notably, its high areal current density of 2 mA cm^−2^ and volumetric capacity of 85 mAh cm^−3^ over 100 cycles outperform previously reported ZIMBs.^[^ ^11^, ^32^, ^47^ ^]^ A detailed study of the electrodeposited cathode revealed the formation of characteristic CoNi‐LDH, highlighting the feasibility of fabricating bifunctional catalysts at the microscale. While the system achieves promising capacity, limited cathodic and anodic stability lead to capacity fade over time. The use of a near‐neutral electrolyte instead of traditional alkaline media adds novelty, enabling high current density and respective areal capacity under milder conditions‐an advantage for integration into compact, on‐chip energy storage systems. Further improvements in areal capacity and cycling stability could be realized through optimized micro‐scale bifunctional catalyst design^[^ ^56^, ^57^, ^58^ ^]^ and enhanced material stability (Zn and the bifunctional catalyst), paving the way for more efficient ZAMB systems. While the present study demonstrates the feasibility of neutral electrolytes for ZAMBs, it is also important to assess their practical application potential in implantable and miniaturized medical implants,^[^ ^59^ ^]^ self‐powered electrochemical sensor (SPES)^[^ ^60^, ^61^ ^]^ *etc*.

## 4 Experimental Section

### Materials

Silver nitrate (AgNO_3_), ammonium chloride (NH_4_Cl), Potassium thiocyanate (KSCN), sodium citrate (C_6_H_5_Na_3_O_7_.2H_2_O) were sourced from Sigma‐Aldrich for the synthesis of porous silver. For the electrodeposition of CoNi‐LDH, cobalt nitrate [Co(NO_3_)_2_], nickel nitrate [Ni(NO_3_)_2_], and for Zn deposition, zinc sulfate (ZnSO_4_. 7H_2_O), sodium sulfate (Na_2_SO_4_), and boric acid (H_3_BO_3_) were also acquired from Sigma‐Aldrich. The gel electrolyte was made by mixing zinc chloride (ZnCl_2_), ammonium chloride (NH_4_Cl), thio urea (CH_4_N_2_S) and polyethylene glycol (PEG), all obtained from Sigma‐Aldrich. Interdigitated gold electrodes (IDEs) with 200 µm line widths and spacings (Model: DRP‐IDEAU200) were purchased from Metrohm U.K. Ltd. All reagents and materials were used as received, without any additional purification steps.

Pt/C (20 wt.%) and triethylene glycol monomethyl ether (TEGMME) were received from Sigma‐Aldrich. Activated carbon (AC) and Super P conductive carbon black were obtained from MTI Corporation. Polyvinylidene fluoride (PVDF) was provided by Alfa Aesar, and dimethylformamide (DMF) was purchased from Severn Biotech Ltd. All materials were used directly as received.

### Electrodeposition of Porous Ag Framework

The Au IDE devices were sequentially cleaned with isopropanol and distilled water, then dried using compressed air. Porous Ag was synthesized using the dynamic hydrogen bubble template method. The electrodeposition solution was prepared by dissolving 0.01 m AgNO_3_, 1.5 m KSCN, and 0.5 m NH_4_Cl, 0.01 m C_6_H_5_Na_3_O_7_.2H_2_O in distilled water. This solution was continuously stirred on a magnetic stirrer for 30 min to ensure uniform mixing. A standard three‐electrode system was employed, using Ag/AgCl as the reference electrode and a platinum wire as the counter electrode. Porous silver was deposited on the Au IDEs at a constant potential of –2 V for 20 s. After the deposition process, the IDE chips were rinsed with distilled water and air‐dried. All electrodeposition steps were carried out using an Auto lab electrochemical workstation.

### Electrodeposition of CoNi‐LDH

CoNi‐LDH was electrochemically deposited on one side (left) of the IDE and porous Ag‐coated IDE. The deposition solution was made by dissolving 0.1 m [Co (NO_3_)_2_] and 0.1 m [Ni (NO_3_)_2_] in the distilled water and stirring the mixture for 10 min to obtain a homogeneous solution. The electrodeposition was performed using a platinum wire as the counter electrode and an Ag/AgCl electrode as the reference. A constant voltage of −0.8 V was applied for 210 s on the Au IDEs and on the porous Ag IDE. After deposition, the electrodes were washed thoroughly with distilled water and left to dry.

### Electrodeposition of Zinc

Zn was electrodeposited on one side of both porous Au IDE devices and flat Au IDE devices using an electrodeposition technique. The Zn solution was prepared by dissolving 12.5 g of sodium sulfate (Na_2_SO_4_), 22.3 g of zinc sulfate heptahydrate (ZnSO_4_. 7H_2_O), and 2 g of boric acid (H_3_BO_3_) in 91 mL of deionized water. This solution was used as the electrolyte for Zn deposition. The electrodeposition process was carried out with a current of −40 mA for 8 s (more deposition time leads to short circuiting by dendrite formation in the edge of the fingers of IDE). After electrodeposition, the IDE devices were rinsed with distilled water and allowed to dry. The same deposition time was maintained for deposition on Ag deposited on the Au IDE chips.

### Preparation of Pt/C Electrode Ink

Pt/C cathode electrode ink was prepared by dispersing 10 wt.% polyvinylidene fluoride (PVDF) in dimethylformamide (DMF), which served as the binder, and combining it with Super‐P carbon black as the conductive component. The 88 mg of Pt/C, 10 mg of Super P, and 2 mg of PVDF were dispersed in 400 mg of triethylene glycol monomethyl ether (TEGMME). The obtained mixture was ground for 30 min in a mortar and pestle to yield a uniformly dispersed electrode ink.

### Printing of Devices

Printing was carried out using a SonoPlot Microplotter Proto equipped with a 20 µm nozzle. The pattern design was created using SonoDraw software. The nozzle was aligned to the starting point of the IDE layout. The printing parameters were set with a feature width of 50 µm in spraying mode. During printing, 10 V was applied for the Pt/C ink.

### Preparation of Gel Electrolyte for Micro‐Batteries

The gel electrolyte for micro‐battery applications was prepared by dissolving 1 g of PVA in 10 g of distilled water, heated at 85 °C in an oil bath for 1 h to form a clear gel. Then, 2.34 m NH_4_Cl, 0.51 m ZnCl_2_, 1000 ppm of CH_4_N_2_S, 1000 ppm of PEG were added to the gel, and the mixture was further heated and stirred at 85 °C for another 2 h to produce a uniform transparent electrolyte.

### Materials Characterization

A scanning electron microscope (SEM) (Zeiss EVO LS15) was used to investigate the low magnification morphology of the devices. The high magnification images, along with EDS mappings, are obtained using a JEOL JSM‐7200F field emission scanning electron microscope (FESEM). Additionally, a stylus profilometer (Bruker DektakXT) was used to examine the devices' surface profiles. X‐ray diffraction (XRD) data are collected using a RIGAKU Smartlab X‐Ray diffractometer, operating at 9 kW power with a Hypix‐3000 detector. High‐resolution XPS spectra are acquired with a PHI Quantera X‐ray photoelectron spectrometer, using a pass energy of 140 eV for survey scans and 26 eV for high‐resolution spectra, with a monochromatic Al Kα source. Raman spectra are obtained using a Renishaw in Via Raman microscope with a 532 nm excitation source (L 50×).

### Electrochemical Tests of Micro‐Batteries

Both ends of the Au IDEs were connected with Cu foil using silver paste and immersed vertically in a cuvette (Fisherbrand Disposable Cuvettes 14955125) filled with gel electrolyte. Both ends of the Cu foil are well separated and properly packed using kepton tape to avoid a short circuit and top of the cuvette was kept open for O_2_ diffusion to the electrolyte. Galvanostatic discharge‐charge (GDC) and cyclic voltammetry (CV) were measured using a Biologic potentiostat. A Neware battery tester was used for long‐term cycle measurements. Electrochemical impedance spectroscopy (EIS) measurements are performed using a Biologic SP‐300 potentiostat. The frequency testing range was 100 KHz to 100 mHz at OCP with a voltage amplitude of 10 mV.

## Conflict of Interest

The authors declare no conflict of interest.

## Supporting information



Supporting Information

## Data Availability

The data that support the findings of this study are available from the corresponding author upon reasonable request.

## References

[1] Y. Zhang , T. Sun , X. Yang , L. Zhou , C. M. J. Tan , M. Lei , H. Bayley , Nat. Chem. Eng. 2024, 1, 691 39620147 10.1038/s44286-024-00136-z PMC11606923

[2] G. Zhang , S. Yang , J. F. Yang , D. Gonzalez‐Medrano , M. Z. Miskin , V. B. Koman , Y. Zeng , S. X. Li , M. Kuehne , A. T. Liu , A. M. Brooks , M. Kumar , M. S. Strano , Sci. Robot. 2024, 9, 93. 10.1126/scirobotics.ade4642 39141708

[3] J. Ma , R. Quhe , W. Zhang , Y. Yan , H. Tang , Z. Qu , Y. Cheng , O. G. Schmidt , M. Zhu , Small 2023, 19, 2300230. 10.1002/smll.202300230 36938705

[4] V. Knap , L. K. Vestergaard , D. I. Stroe , Energies (Basel) 2020, 13, 4097.

[5] J. Fan , C. Liu , N. Li , L. Yang , X.‐G. Yang , B. Dou , S. Hou , X. Feng , H. Jiang , H. Li , W.‐L. Song , L. Sun , H.‐S. Chen , H. Gao , D. Fang , Nature 2025, 641, 639. 40369137 10.1038/s41586-025-08785-7

[6] Y. Ma , S. Wang , Z. S. Wu , Nanomicro Lett. 2025, 17, 105. 39775337 10.1007/s40820-024-01625-9 PMC11711423

[7] B. Ke , X. Wang , Proc. Natl. Acad. Sci. USA 2025, 122, 2415693122.

[8] A. D. Refino , C. Eldona , R. F. H. Hernandha , E. Adhitama , A. Sumboja , E. Peiner , H. S. Wasisto , Commun. Mater. 2024, 5, 22.

[9] H. Yoo , M. Mahato , W. Oh , J. Ha , H. Han , C. W. Ahn , I. K. Oh , Biosens. Bioelectron. 2024, 260, 116419. 38830292 10.1016/j.bios.2024.116419

[10] Y. Fan , I. Pinnock , X. Hu , T. Wang , Y. Lu , R. Li , M. Wang , I. P. Parkin , M. De Volder , B. D. Boruah , Nano Lett. 2024, 24, 10874. 39163512 10.1021/acs.nanolett.4c02539 PMC11378291

[11] N. Naresh , Y. Fan , Y. Zhu , T. Wang , S. Li , I. P. Parkin , M. De Volder , B. D. Boruah , Adv. Funct. Mater. 2025, 35, 43.

[12] J. Zhang , Y. Huang , Q. Yang , V. Venkatesh , M. Synodis , J. H. Pikul , S. A. Bidstrup Allen , M. G. Allen , ACS Appl. Mater. Interfaces 2023, 15, 6807. 36700920 10.1021/acsami.2c19970

[13] H. Zhang , Z. Qu , H. Tang , X. Wang , R. Koehler , M. Yu , C. Gerhard , Y. Yin , M. Zhu , K. Zhang , O. G. Schmidt , ACS Energy Lett. 2021, 6, 2491.

[14] G. Liu , Z. Ma , G. Li , W. Yu , P. Wang , C. Meng , S. G. Guo Liu , Z. Ma , G. Li , W. Yu , P. Wang , C. Meng , S. Guo , ACS Appl. Mater. Interfaces 2023, 15, 13073. 36866775 10.1021/acsami.2c22233

[15] Y. Ma , S. Wang , Z. S. Wu , Nanomicro Lett 2025, 17, 105. 39775337 10.1007/s40820-024-01625-9 PMC11711423

[16] Y. Li , A. Huang , L. Zhou , B. Li , M. Zheng , Z. Zhuang , C. Chen , C. Chen , F. Kang , R. Lv , Nat. Commun. 2024, 15, 8365. 39333097 10.1038/s41467-024-52494-0 PMC11436649

[17] K. Wang , P. Pei , Z. Ma , H. Chen , H. Xu , D. Chen , X. Wang , J. Mater. Chem. A Mater. 2015, 3, 22648.

[18] H. Zhang , Z. Qu , H. Tang , X. Wang , R. Koehler , M. Yu , C. Gerhard , Y. Yin , M. Zhu , K. Zhang , O. G. Schmidt , ACS Energy Lett. 2021, 6, 2491.

[19] G. Liu , Z. Ma , G. Li , W. Yu , P. Wang , C. Meng , S. Guo , ACS Appl. Mater. Interfaces 2023, 15, 13073. 36866775 10.1021/acsami.2c22233

[20] W. Ling , Q. Yang , F. Mo , H. Lei , J. Wang , Y. Jiao , Y. Qiu , T. Chen , Y. Huang , Energy Storage Mater. 2022, 51, 453.

[21] Y. Zhu , D. Zheng , X. Xing , S. Wu , X. Guo , J. Liu , X. Guo , J. Zhou , Y. Jiao , B. Zeng , N. Wang , L. Wan , H. Zhang , S. Liu , J. Phys. Chem. C 2024, 128, 7007.

[22] S. Cherevko , X. Xing , C. H. Chung , Electrochem. Commun. 2010, 12, 467.

[23] N. S. Gultom , M. Z. Silitonga , K. X. Hu , Y. C. Zhou , D. H. Kuo , J. Alloys Compd. 2023, 955, 170232.

[24] Y. Fan , N. Naresh , Y. Zhu , M. Wang , B. D. Boruah , ACS Nano. 2025, 19, 13. 40132082 10.1021/acsnano.5c00917 PMC11984303

[25] S. R. Pattanayak , P. Thakur , T. N. Narayanan , Cell Rep. Phys. Sci. 2025, 6, 5.

[26] B. A. Brandes , Y. Krishnan , F. L. Buchauer , H. A. Hansen , J. Hjelm , Nat. Commun. 2024, 15, 7336. 39187503 10.1038/s41467-024-51605-1 PMC11347700

[27] Z. Zhang , Z. Zhang , C. Chen , R. Wang , M. Xie , S. Wan , R. Zhang , L. Cong , H. Lu , Y. Han , W. Xing , Z. Shi , S. Feng , Nat Commun. 2024, 15, 2556. 38519497 10.1038/s41467-024-46872-x PMC10960042

[28] H. Wang , L. Kang , K. Wang , M. Wei , P. Pei , Y. Zuo , B. Liang , Adv. Funct. Mater. 2024, 34, 44.

[29] C. Wang , J. Li , Z. Zhou , Y. Pan , Z. Yu , Z. Pei , S. Zhao , L. Wei , Y. Chen , EnergyChem 2021, 3, 100055.

[30] J. Zhang , M. G. Allen , EES Batteries 2025, 1, 878.

[31] F. W. Thomas Goh , Z. Liu , T. S. A. Hor , J. Zhang , X. Ge , Y. Zong , A. Yu , W. Khoo , J. Electrochem. Soc. 2014, 161, A2080.

[32] N. Naresh , Y. Zhu , J. Luo , Y. Fan , T. Wang , K. Raju , M. De Volder , I. P. Parkin , B. D. Boruah , Adv. Funct. Mater. 2024, 35, 3.

[33] M. Fantauzzi , F. Secci , M. Sanna Angotzi , C. Passiu , C. Cannas , A. Rossi , RSC Adv. 2019, 9, 19171. 35685202 10.1039/c9ra03488a PMC9128503

[34] R. Jana , R. Bhunia , S. Paramanik , K. Giri , A. Chowdhury , Adv. Funct. Mater. 2024, 35, 2.

[35] Q. Wu , X. Fan , B. Shan , L. Qi , X. Quan , Y. Liu , Nat. Commun. 2025, 16, 3479. 40216792 10.1038/s41467-025-58811-5 PMC11992037

[36] T. Liu , L. Wang , B. Chen , H. Liu , S. Wang , Y. Feng , J. Zhang , Y. Yin , M. D. Guiver , Angew. Chem. ‐ Int. Ed. 2025, 64, 11. 10.1002/anie.202421869 39810745

[37] B. Laïk , M. Richet , N. Emery , S. Bach , L. Perrière , Y. Cotrebil , V. Russier , I. Guillot , P. Dubot , ACS Omega 2024, 9, 40707. 39371995 10.1021/acsomega.4c05082 PMC11447992

[38] I. Saric , R. Peter , M. Petravic , J. Phys. Chem. C 2016, 120, 22421.

[39] S. Sheng , Y. Ren , S. Yang , Q. Wang , P. Sheng , X. Zhang , Y. Liu , ACS Omega 2020, 5, 17703. 32715257 10.1021/acsomega.0c02301 PMC7377325

[40] G. Niaura , A. K. Gaigalas , V. L. Vilker , J. Phys. Chem. B 1997, 101, 9250.

[41] W. Yan , D. Wang , G. G. Botte , Electrochim. Acta 2012, 61, 25.

[42] M. Gao , Y. Li , J. Yang , Y. Liu , Y. Liu , X. Zhang , S. Wu , K. Cai , Chem. Eng. J. 2022, 429, 132423.

[43] P. K. Ray , R. Mohanty , K. Parida , J. Energy Storage 2023, 72, 108335.

[44] Q. Wu , F. Li , H. Sheng , Y. Qi , J. Yuan , H. Bi , W. Li , E. Xie , W. Lan , ACS Appl. Mater. Interfaces 2024, 16, 23241. 38669688 10.1021/acsami.4c01533

[45] Z. Liu , X. Huang , X. Liu , J. Liu , M. Wang , T. Ding , L. Yan , Z. Zhang , G. Shi , Small 2024, 21, 2. 10.1002/smll.202408566 39498700

[46] X. Hu , L. Zhang , S. Li , J. Chen , B. Zhang , Z. Zheng , H. He , S. Luo , A. Xie , New J. Chem. 2022, 46, 19491.

[47] Y. Zhu , N. Naresh , X. Liu , J. Luo , Y. Fan , M. Cao , B. Li , M. Wang , B. D. Boruah , Small 2024, 21, 7. 10.1002/smll.202405733 PMC11840458 39400434

[48] H. S. Min , B. Y. Park , L. Taherabadi , C. Wang , Y. Yeh , R. Zaouk , M. J. Madou , B. Dunn , J. Power Sources 2008, 178, 795.

[49] S. Bi , F. Wan , S. Huang , X. Wang , Z. Niu , ChemElectroChem 2019, 6, 3933.

[50] Y. Zeng , Y. Meng , Z. Lai , X. Zhang , M. Yu , P. Fang , M. Wu , Y. Tong , X. Lu , Adv. Mater. 2017, 29, 44. 10.1002/adma.201702698 28991385

[51] L. He , T. Hong , X. Hong , X. Liao , Y. Chen , W. Zhang , H. Liu , W. Luo , L. Mai , Energy Technol. 2019, 7, 9.

[52] M. Kotobuki , Y. Suzuki , H. Munakata , K. Kanamura , Y. Sato , K. Yamamoto , T. Yoshida , Electrochim. Acta 2011, 56, 1023.

[53] X. Wang , S. Zheng , F. Zhou , J. Qin , X. Shi , S. Wang , C. Sun , X. Bao , Z.‐S. Wu , Natl. Sci. Rev. 2019, 7, 1. 10.1093/nsr/nwz070 PMC8288951 34692018

[54] X. Wang , H. Huang , F. Zhou , P. Das , P. Wen , S. Zheng , P. Lu , Y. Yu , Z.‐S. Wu , Nano Energy 2021, 82, 105688.

[55] B. Zhao , S. Wang , Q. Yu , Q. Wang , M. Wang , T. Ni , L. Ruan , W. Zeng , J. Power Sources 2021, 504, 230076.

[56] H. Shi , S. Gao , X. Liu , Y. Wang , S. Zhou , Q. Liu , L. Zhang , G. Hu , Small 2024, 20, 25. 10.1002/smll.202309557 38705855

[57] J. Zhou , M. Zhang , Y. Lin , J. Xu , C. Pan , Y. Lou , Y. Zhang , Y. Wang , Y. Dong , Y. Zhu , J. Zhang , Z. Lin , Nano Energy 2024, 125, 109529.

[58] J. Xiang , P. Wang , P. Li , M. Zhou , G. Yu , Z. Jin , Angew. Chem. ‐ Int. Ed. 2025, 64, 21. 10.1002/anie.202500644 40033984

[59] L. Li , D. Li , Y. Wang , T. Ye , E. He , Y. Jiao , L. Wang , F. Li , Y. Li , J. Ding , K. Liu , J. Ren , Q. Li , J. Ji , Y. Zhang , Adv. Mater. 2023, 35, 32. 10.1002/adma.202302997 37159396

[60] J. Zhu , Y. Xiao , W. Hu , Q. Cui , Y. Yuan , X. Peng , W. Wen , X. Zhang , S. Wang , Anal. Chem. 2024, 96, 1852. 38279192 10.1021/acs.analchem.3c03423

[61] Q. Wang , D. Jiang , X. Du , X. Shan , W. Wang , H. Shiigi , Z. Chen , Analyst 2024, 149, 2291. 38511612 10.1039/d4an00200h

