# Supplementary material for: Microscale Near‐Neutral Zinc–Air Battery on Interdigitated Electrode Chips for High Current Operation
Source: Small Methods. 2025 Nov 10;9(12):e01562. doi: 10.1002/smtd.202501562 (PMC12716186; doi:10.1002/smtd.202501562)
Supplement: Supplementary file 1 — Supporting Information [file SMTD-9-e01562-s001.pdf]

Supplementary Information

Microscale Near-Neutral Zinc–Air Battery on Interdigitated Electrode Chips for High Current Operation

Subhra R. Pattanayak,[1] *Nibagani Naresh,*[2] *Yujia Fan,*[2] *Yijia Zhu,*[2] *Tharangattu N. Narayanan,*[1,*] *Buddha Deka Boruah*[2,*]

[1]Tata Institute of Fundamental Research Hyderabad, Serilingampally Mandal, Hyderabad 500046, India.

[2]Institute for Materials Discovery, University College London, London WC1E 7JE, United Kingdom

Corresponding authors:

Prof. Tharangattu N. Narayanan

Email: tnn@tifrh.res.in

Dr. Buddha Deka Boruah

Email: b.boruah@ucl.ac.uk

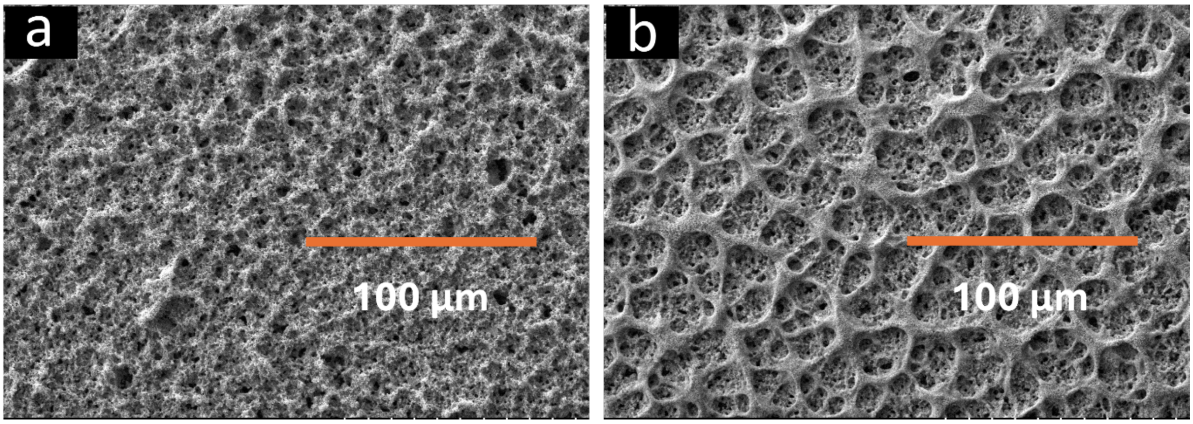

Figure S1. SEM images of porous silver at (a) -2 V for 10 seconds and (b) -2 V for 20 seconds

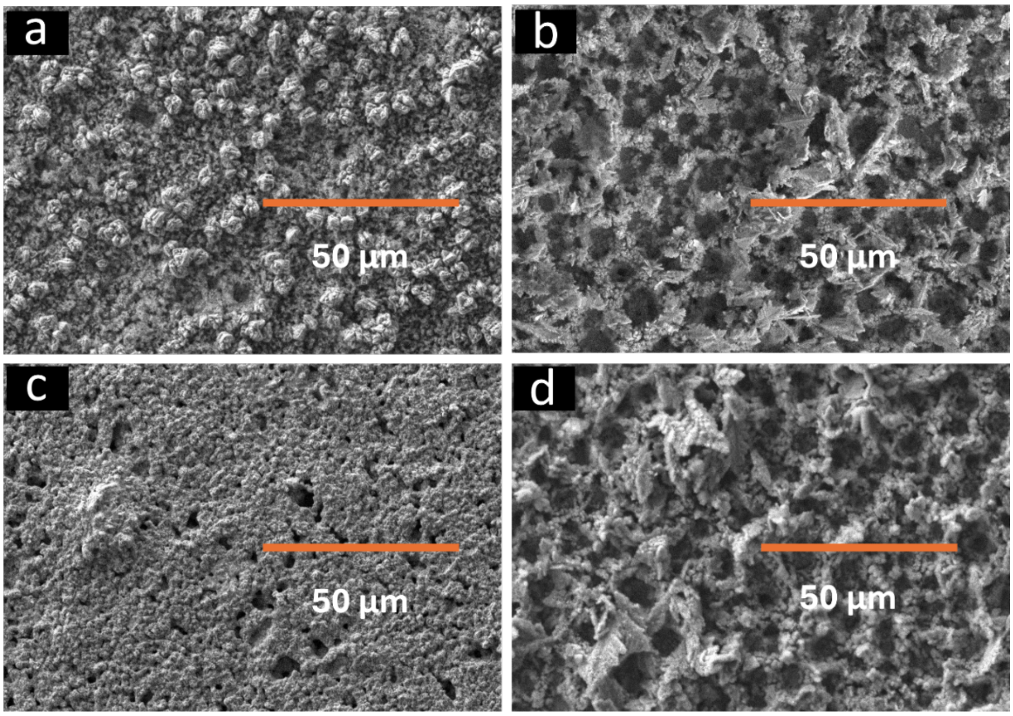

Figure S2. SEM images of Zn at – 40 mA for 7 seconds on (a) Au and (b) Ag on Au. After dipping in neutral electrolyte for 1 hour showing (a) Zn loss from Au surface, (d) Zn stability on Ag/Au surface.

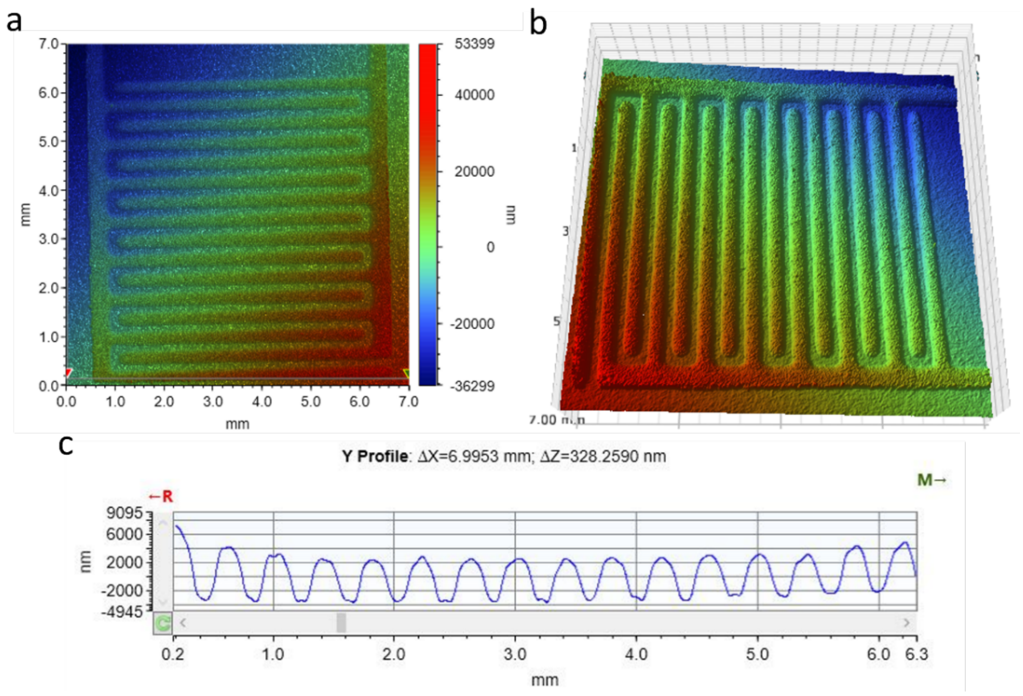

Figure S3. (a, b) 2D and 3D profilometer images of the porous Ag on Au IDE chips, depicting electrode height profiles, (c) showing an average thickness of ~ 3.5 µm, excluding the flat Au IDEs thickness of ~ 4 µm.

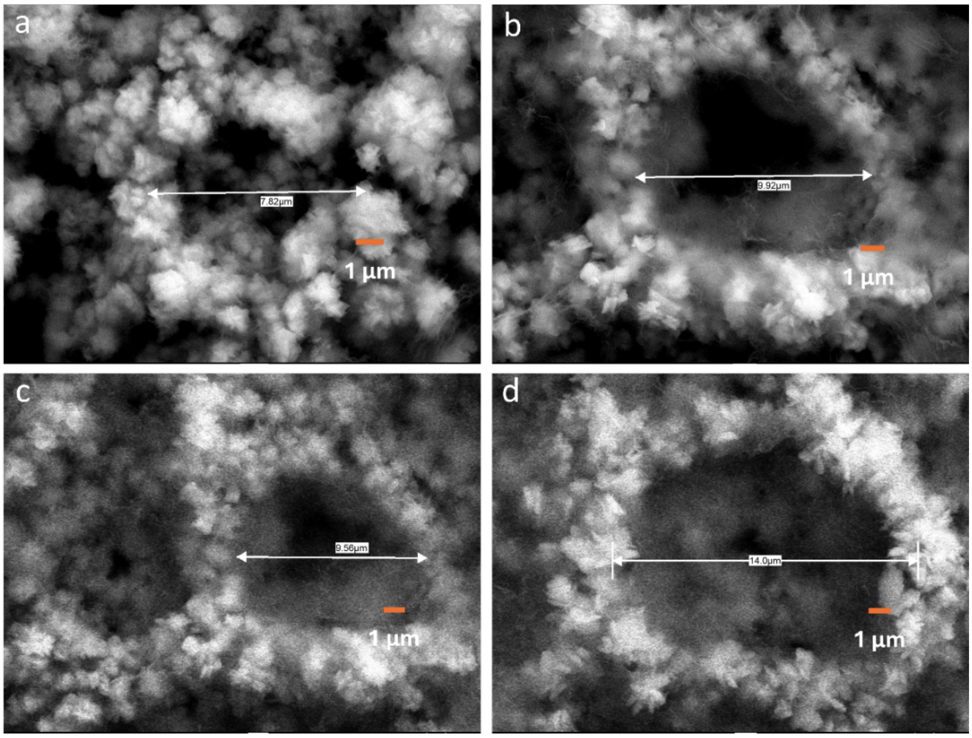

Figure S4. The SEM images of Zn deposited on Ag having pore size of (a) 7.82 µm (b) 9.92 µm (c) 9.56 µm (d) 14 µm.

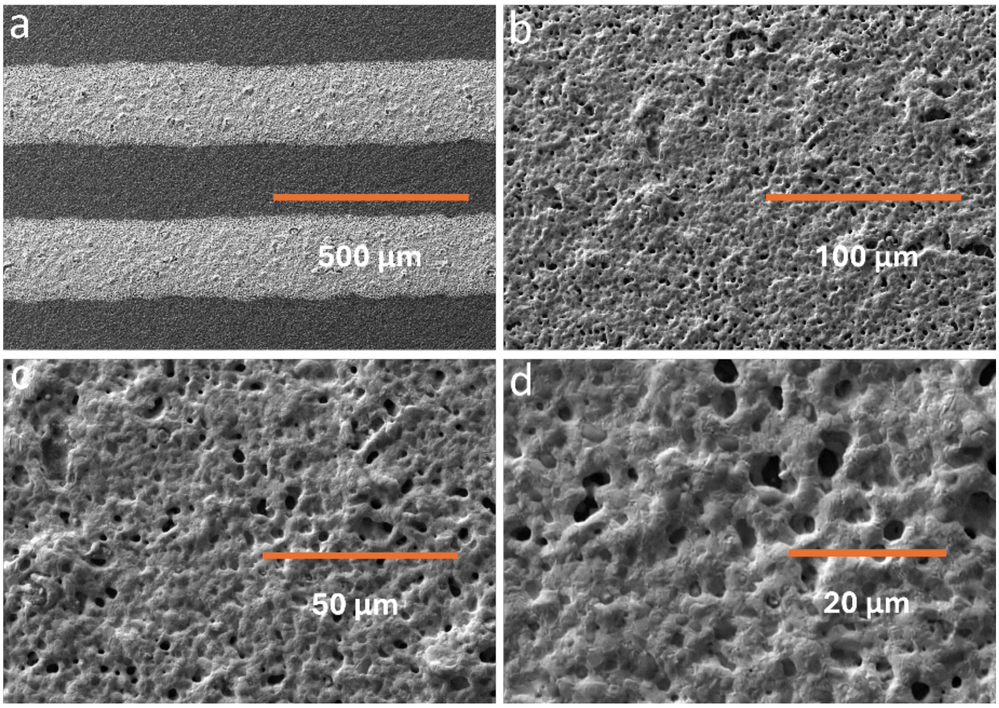

Figure S5. SEM images of Flat Au surface on the IDE chips at different magnifications, (a) interdigitated fingers at low magnification, (b,c,d) high magnified images of Au IDE chip.

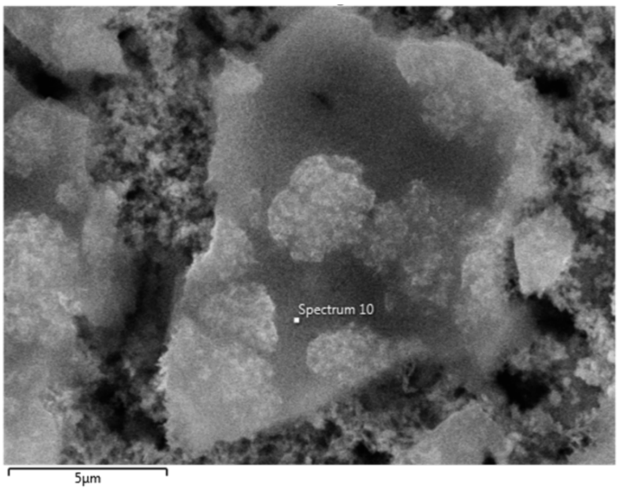

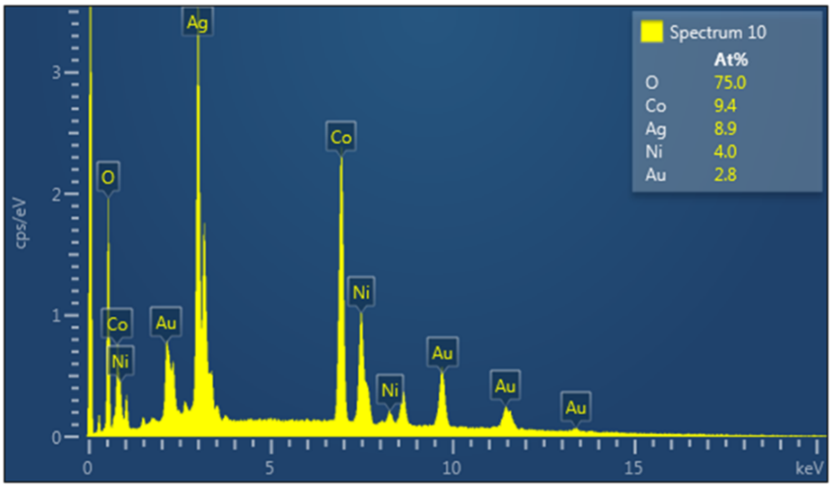

Figure S6. EDS point analysis showing Ag, Co, Ni on the flakish feature of cathodic side of CN-ZAMB. The Co/Ni atomic percentage ratio is found to be more than 2 times.

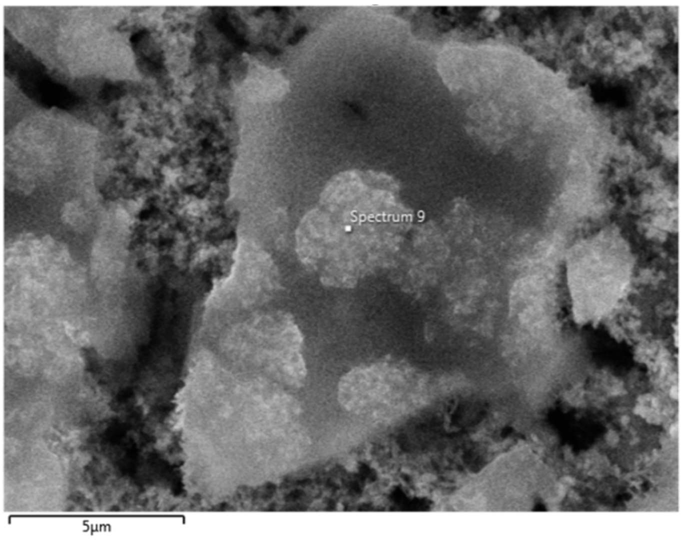

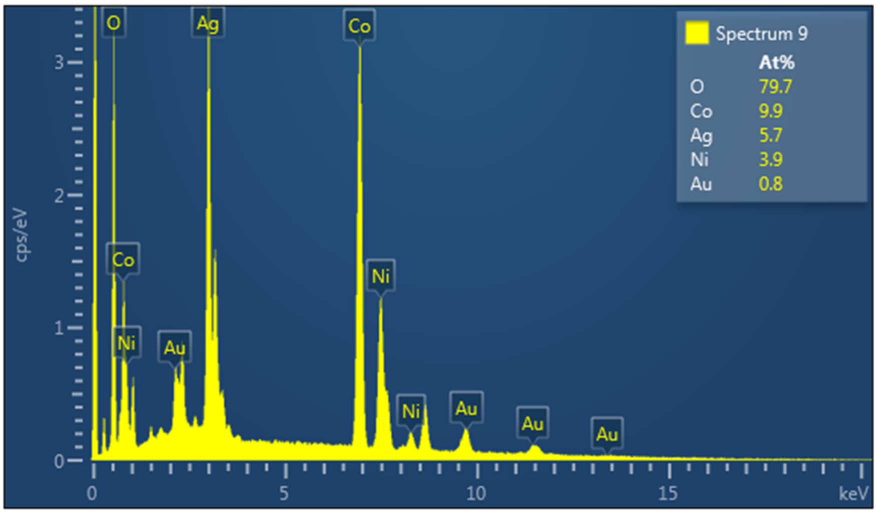

Figure S7. EDS point analysis showing Ag, Co, Ni on the flakish feature of cathodic side of CN-ZAMB. Here the point on the flower like feature also shows Co/Ni atomic percentage ratio more than 2 times.

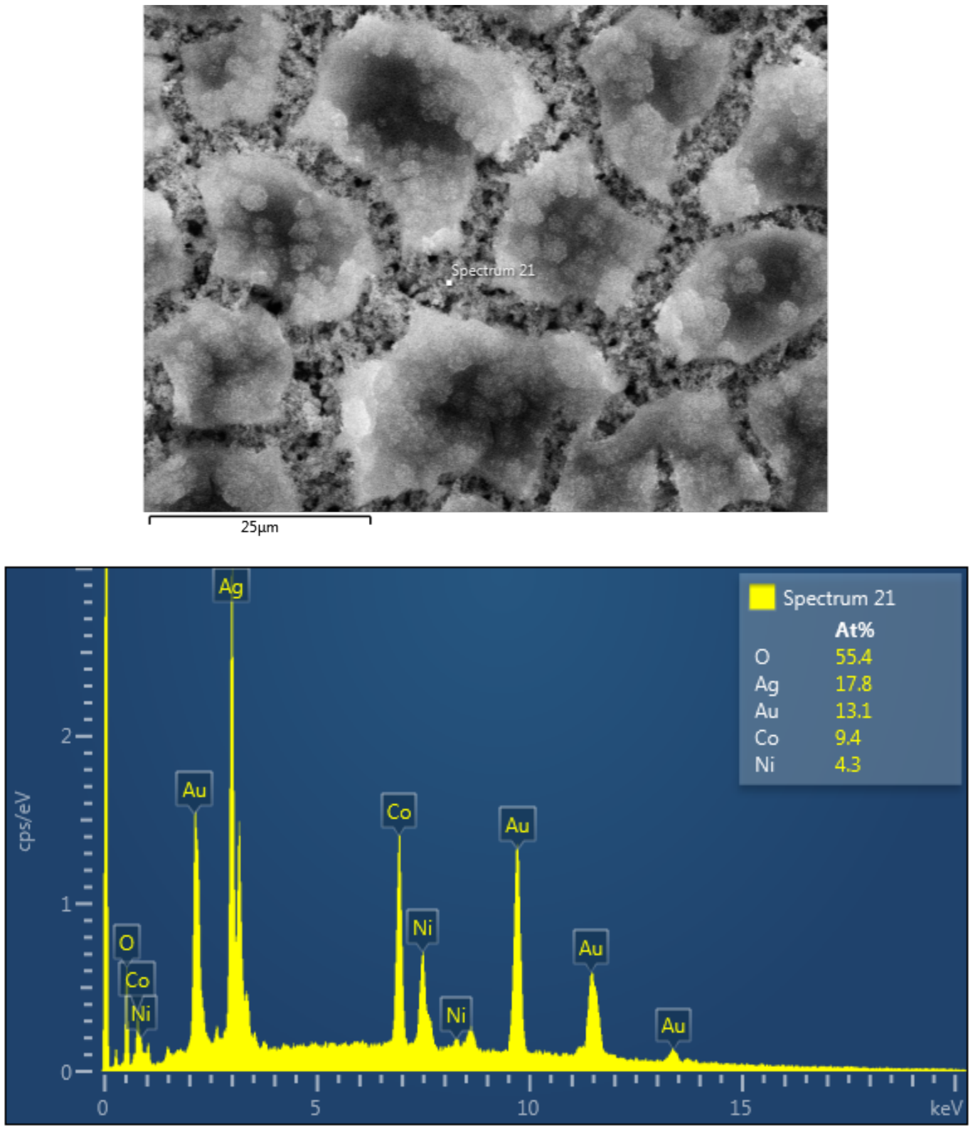

Figure S8. EDS point analysis showing Ag, Co, Ni on the non-flakish feature of cathodic side of CN-ZAMB. This also shows Co/Ni atomic percentage ratio more than 2 times.

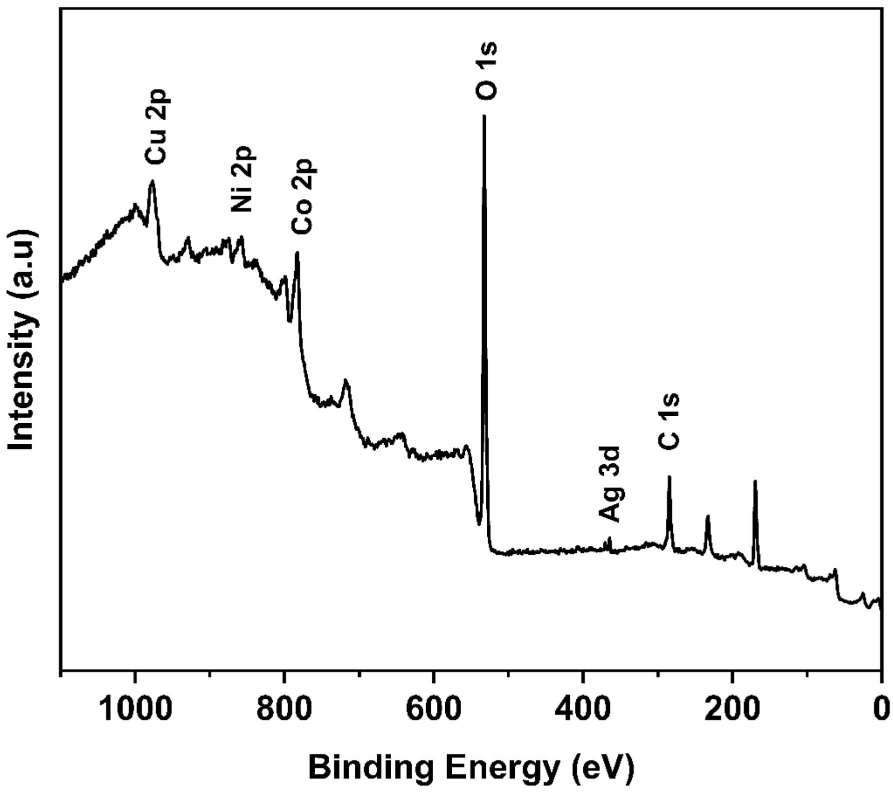

Figure S9. Survey spectra of Co(II)/Ni(III) deposited on Ag (on the Cu substrate). This survey spectra confirms the presence of Cu 2p, Ag 3d, Co 2p, Ni 2p, O 1s, C 1s in the sample.

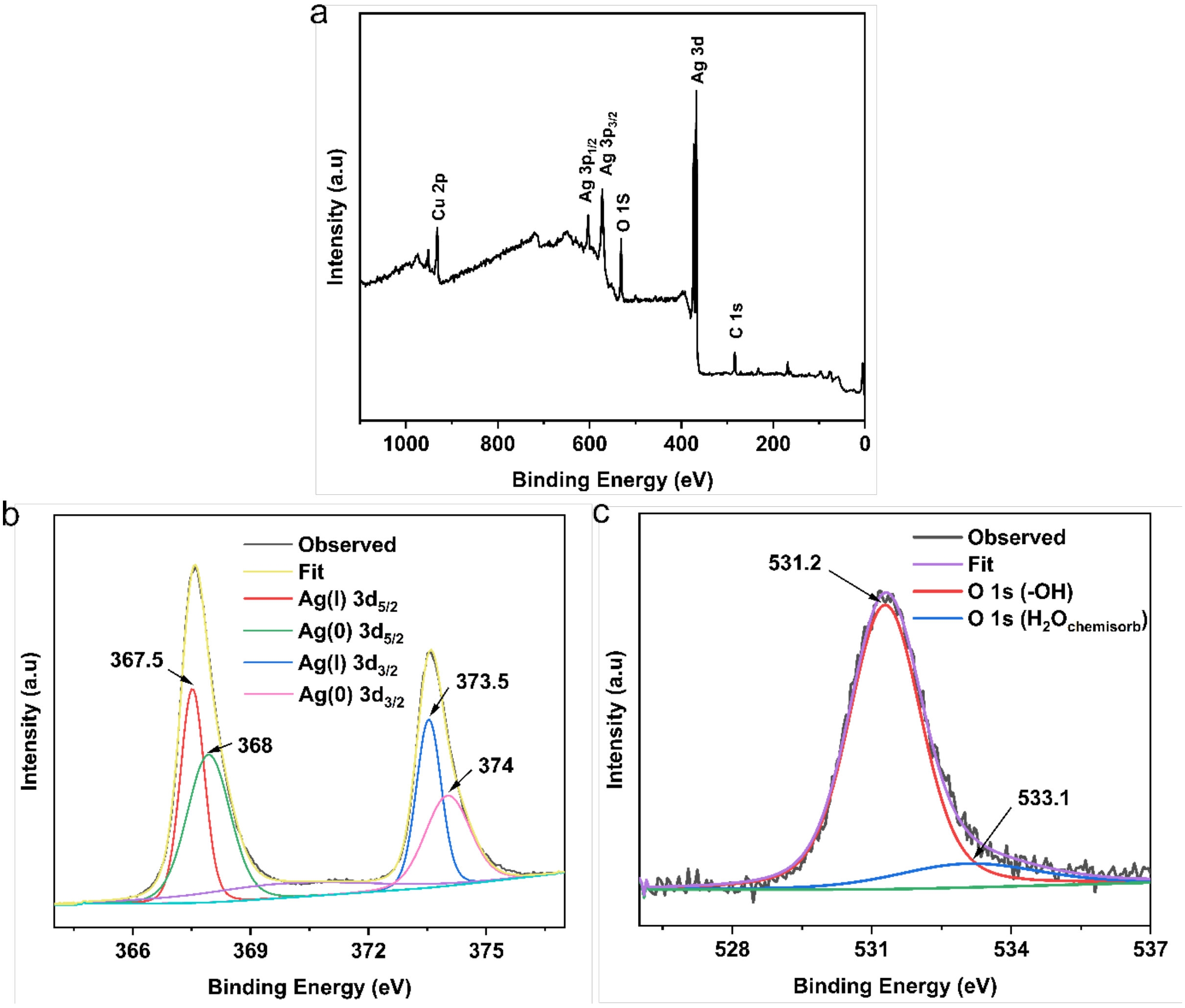

Figure S10. XPS spectra of Ag deposited on the Cu substrate. The survey spectra in (a) confirms the presence of Cu 2p, Ag $2p_{3/2}$, Ag $2p_{1/2}$, O 1s, Ag 3d, C 1s in the sample. The XPS spectra on (b) shows metallic and surface oxidised Ag demonstrating Ag(0) $3d_{5/2}$, $3d_{3/2}$ [1], Ag(I) $3d_{5/2}$, $3d_{3/2}$[2]

. The XPS spectra on (c) O 1s spectra at 531.2 eV shows the presence of surface hydroxyl groups and at 533.1 shows chemisorbed H_2O containing species. [3]

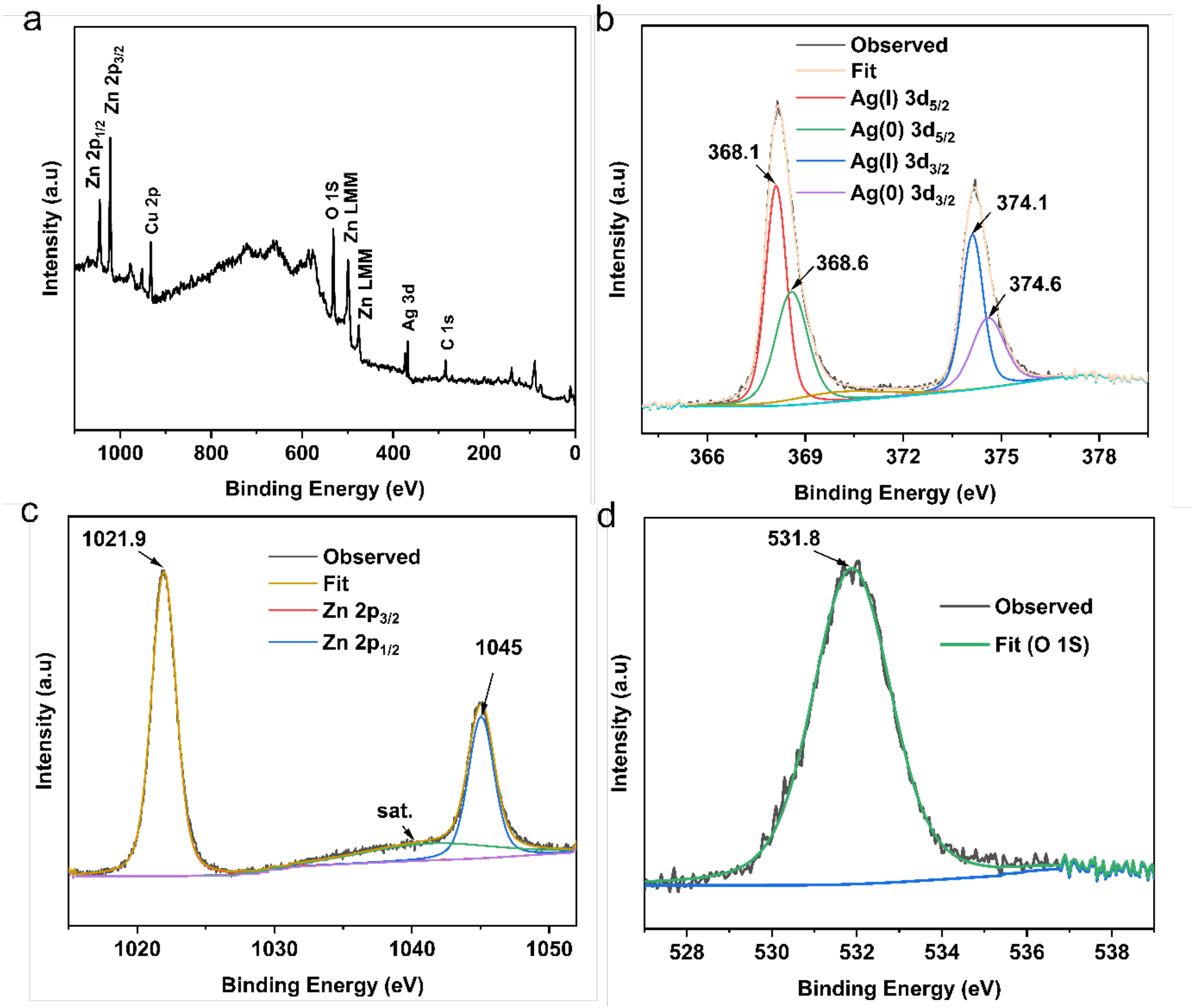

Figure S11. XPS spectra of Zn deposited sample on Ag (on the Cu substrate). The survey spectra in (a) confirms the presence of Cu 2p, Cu LMM, Ag 3d, Zn $2p_{3/2}$, Zn $2p_{1/2}$, O 1s, C 1s in the sample. The XPS spectra on (b) shows metallic and surface oxidised Ag demonstrating Ag(0) $3d_{5/2}$, $3d_{3/2}$, Ag(I) $3d_{5/2}$, $3d_{3/2}$.

The XPS spectra on (c) shows metallic Zn $2p_{3/2}$, Zn $2p_{1/2}$ at 1021.9 eV and 1045 eV respectively having 23.1 eV difference in binding energy as reported from literature[4,5] and (d) O 1s spectra at 531.8 eV shows the presence of surface hydroxyl groups and chemisorbed oxygen containing species.[6]

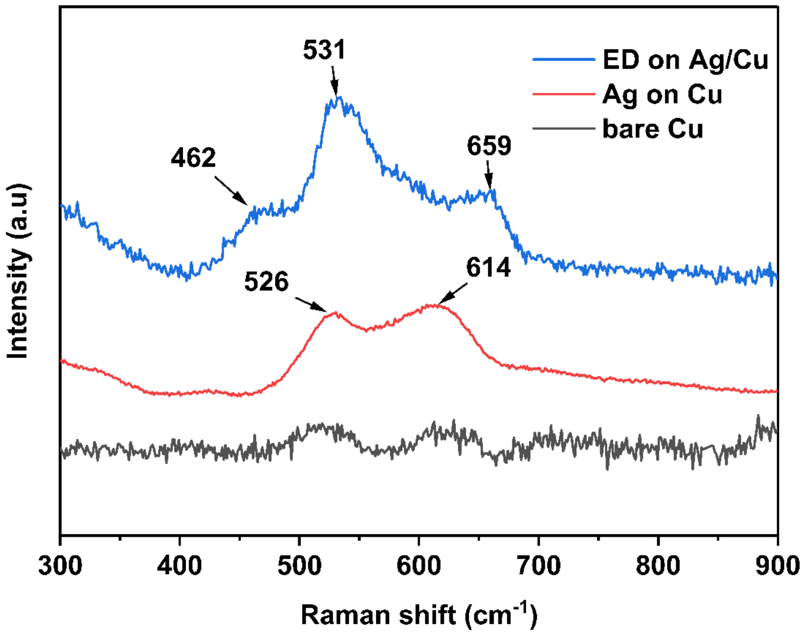

Figure S12. Raman spectra of the samples. Ag on Cu induces SERS, enhancing the weak CuO peaks at 526 and 614 cm^{-1}. The new peaks generated at 462 cm^{-1} and 531 cm^{-1} come from Ni-OH/Co-OH and Ni-O/Co-O stretching modes of NiCo-LDH. There are superposition of various peaks and the peak at 659 cm^{-1} belong to a different mode activated by Co/Ni deposition on Ag/Cu.

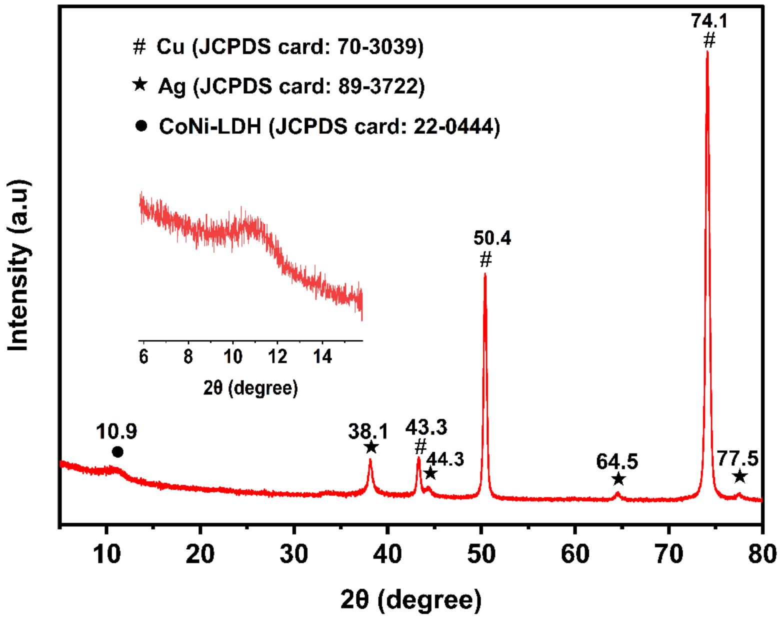

Figure S13. XRD peaks of Cu (JCPDS card No. 70-3039), Ag (JCPDS card No. 89–3722), major peak of CoNi-LDH (JCPDS card No. 22-0444) at 10.9

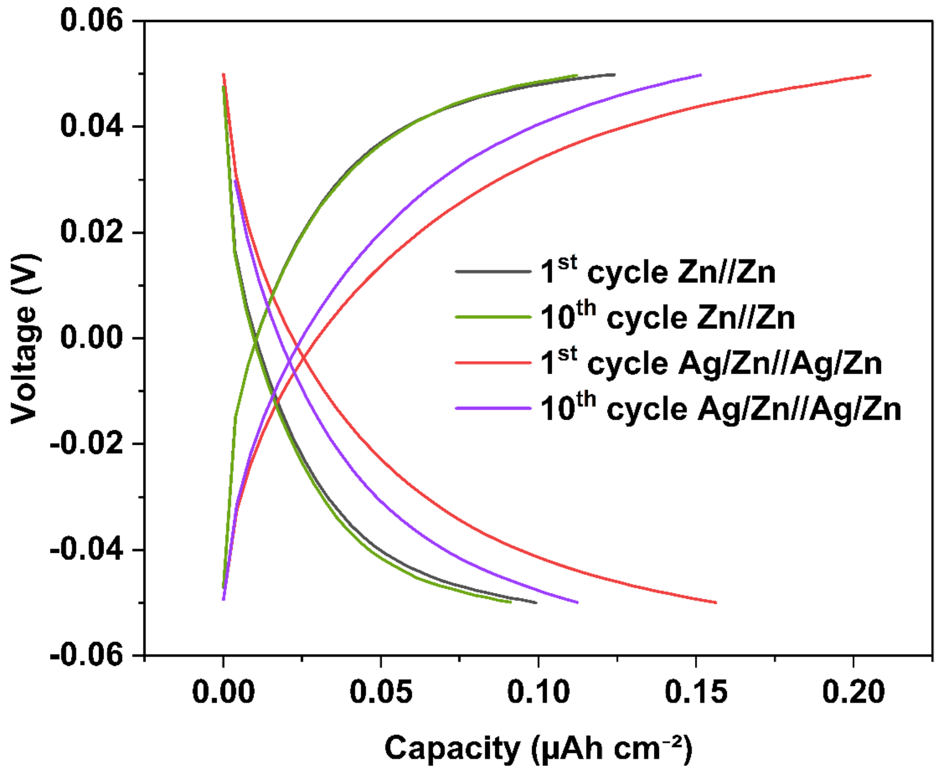

Figure S14. Cyclic voltammetry was performed on symmetric cells (Zn//Zn and Ag/Zn//Ag/Zn) over a potential range of 0.4 V to –0.4 V for 10 cycles. This shows that the absolute capacity is better for Ag/Zn than Zn symmetric cells after 10 cycles.

Calculation of e$^-$ transfer in ORR:

The oxygen reduction reaction (ORR) process was studied in detail for the 20% Pt/C catalyst that's used to microplot the cathode side of the interdigitated chips (IDE). The ORR on the 20 wt% Pt/C was investigated by hydrodynamic voltammetry and Koutecky–Levich (K–L) linearization. Currents were normalized to the geometric disk area, and cathodic values were treated as negative; therefore, we plotted 1/(-j) versus 1/√ω, where ω = 2πN/60 (rad s^{-1}) and N is the rotation rate in rpm. The data were fitted to the K–L relation.[7]

$$1/j = 1/j_k + 1/\,(B\ \omega^{1/2}\}),\ B = 0.62\ n\ F\ D^{2/3}_{O2}\ v^{-1/6}\ C_{O2}$$

where j is the measured current density (mA cm^{-2}), j_k is the kinetic current density, n is the apparent electron-transfer number, F is Faraday's constant (96485 C mol^{-1}), D_{O2} is the O_2 diffusion coefficient (cm^2 s^{-1}), ν is the kinematic viscosity (cm^2 s^{-1}), and C_{O2} is the dissolved

O_2 concentration (mol cm^{-3}). Linear fit of 1/(-j) vs 1/√ω yielded the slope m and n can be obtained *via* the following equation:

$$n = 1 / [1000 \cdot m \cdot (0.62\ n\ F\ D^{2/3}_{O2}\ v^{-1/6}\ C_{O2})]$$

From the RDE plot obtained at different rotation speed (Figure S15), the slope of the linear fit of 1/(-j) vs 1/√ω (Figure S16) is obtained as 2.57 at 0.63 V *vs* RHE and apparent electron-transfer number (n) value is obtained to be ~ 3.5 that indicates Pt/C involves major direct 4 electron transfer in the half cell study, as reported by previous studies.[8] However, it is challenging to get accurate idea about the electron transference number for CN catalyst as that involves electrodeposition process on Au IDE, but the similar performance of the cells constructed from the Pt/C catalyst indicates the major direct 4 e^- transfer involved ORR happening at the electrode.

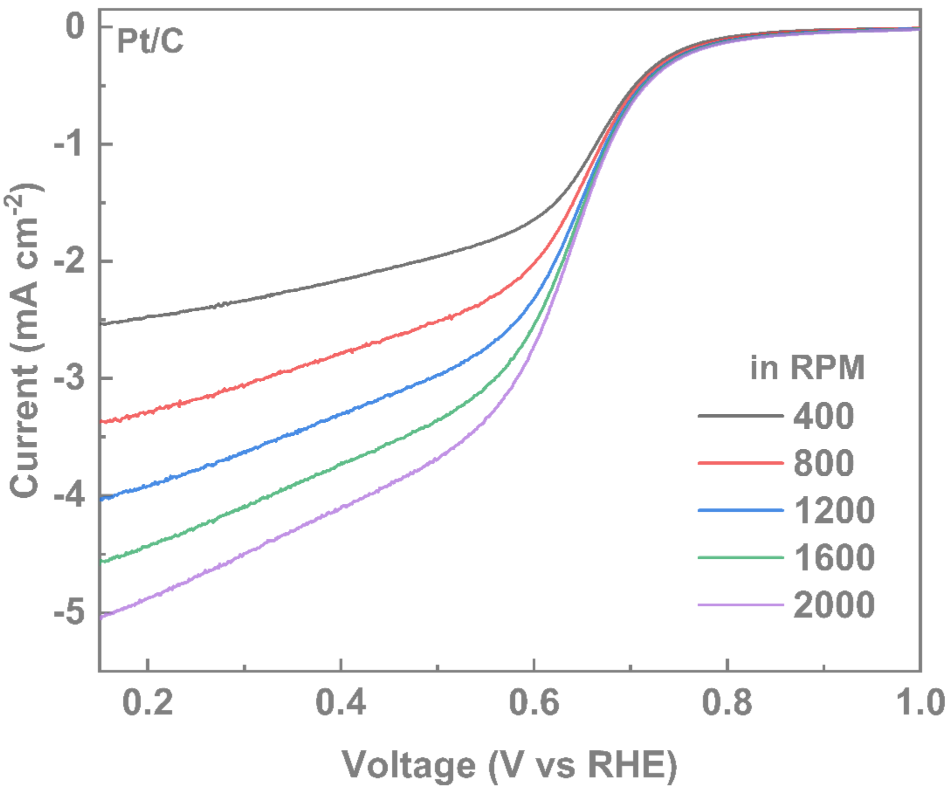

Figure S15. RDE plot obtained at different rotation speed

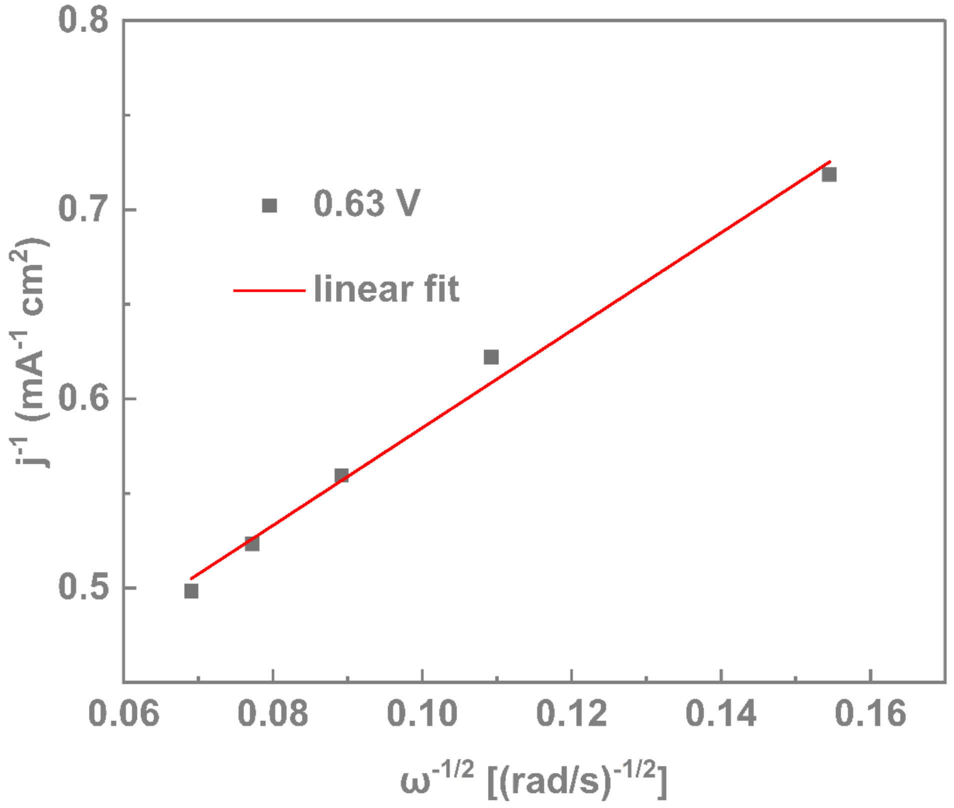

Figure S16. K-L plot showing linear fit having R^2 value to be ~0.98.

We have measured the open circuit voltage of the CN-ZAMB and Pt-ZAMB and they are found to be ~ 0.95 V. The images of the multimeter connected to the micro–air batteries are shown below.

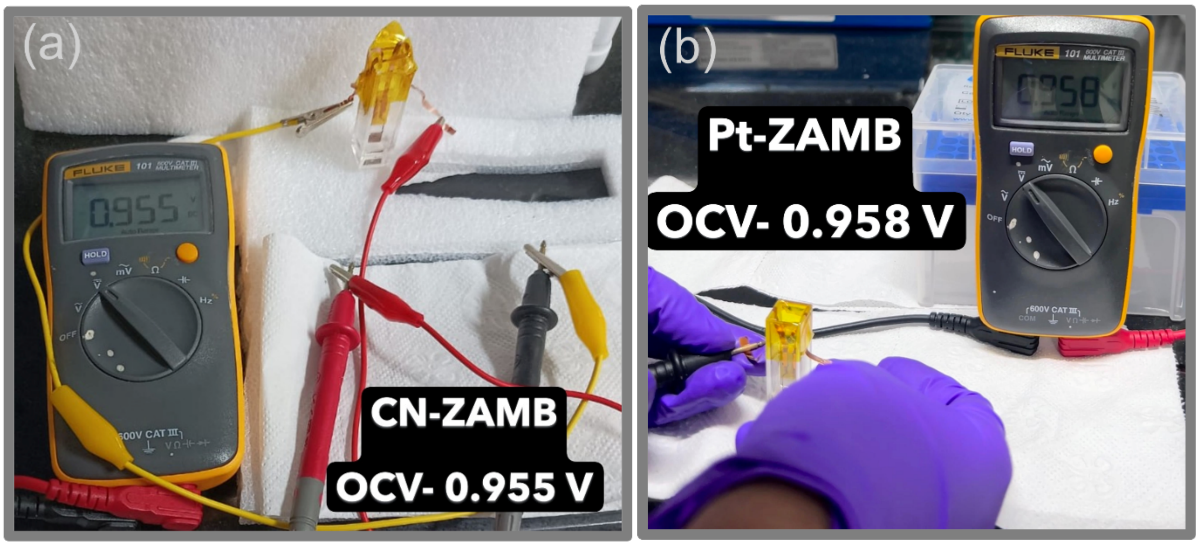

Figure S17. Multimeter readings showing open circuit voltage of (a) the CN-ZAMB and (b) Pt-ZAMB.

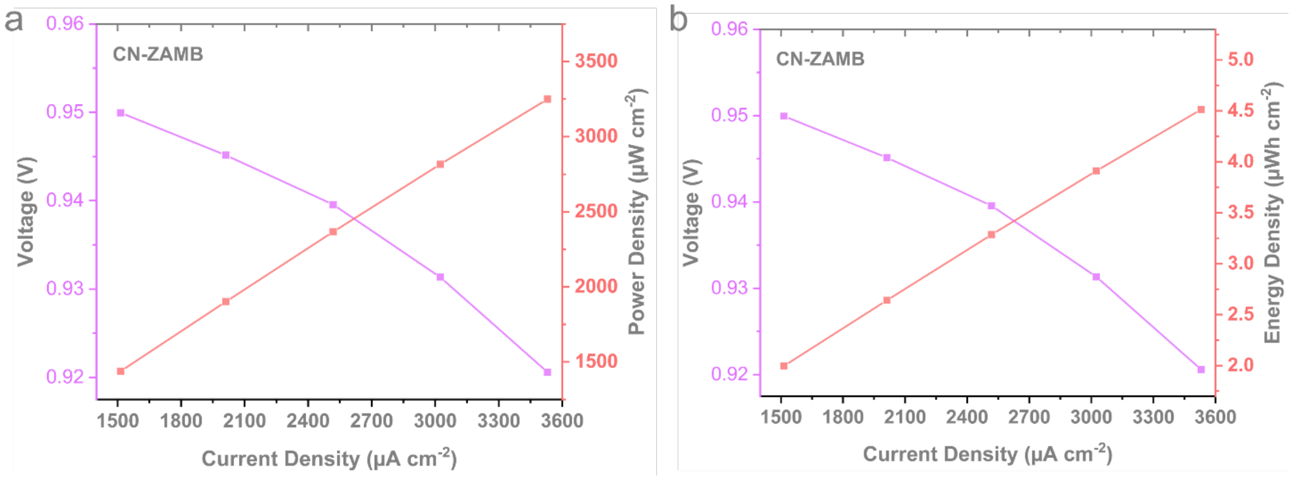

Figure S18. The discharge polarization curves with corresponding (a) power density and (b) Energy density for CN-ZAMB

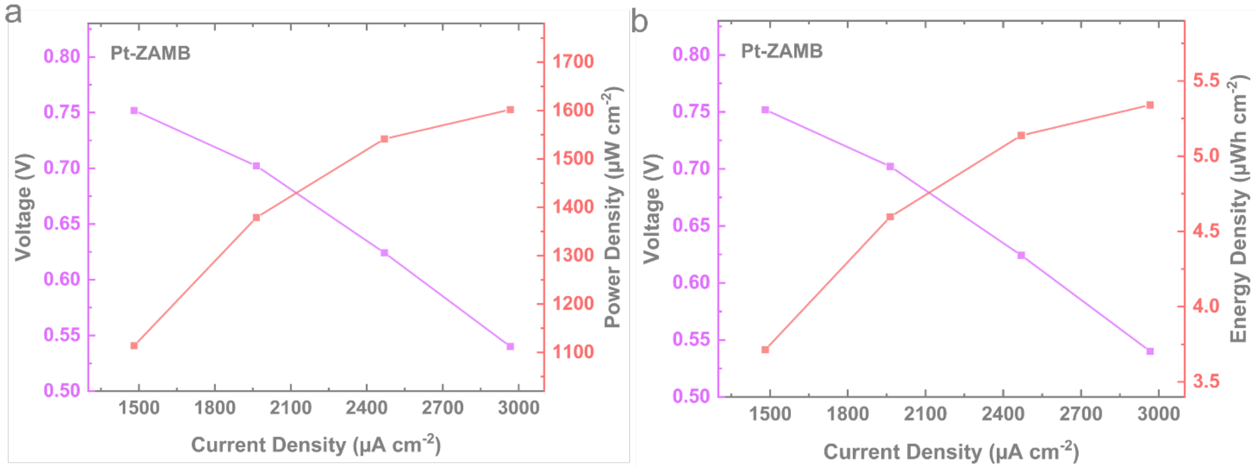

Figure S19. The discharge polarization curves with corresponding (a) power density and (b) Energy density for Pt-ZAMB

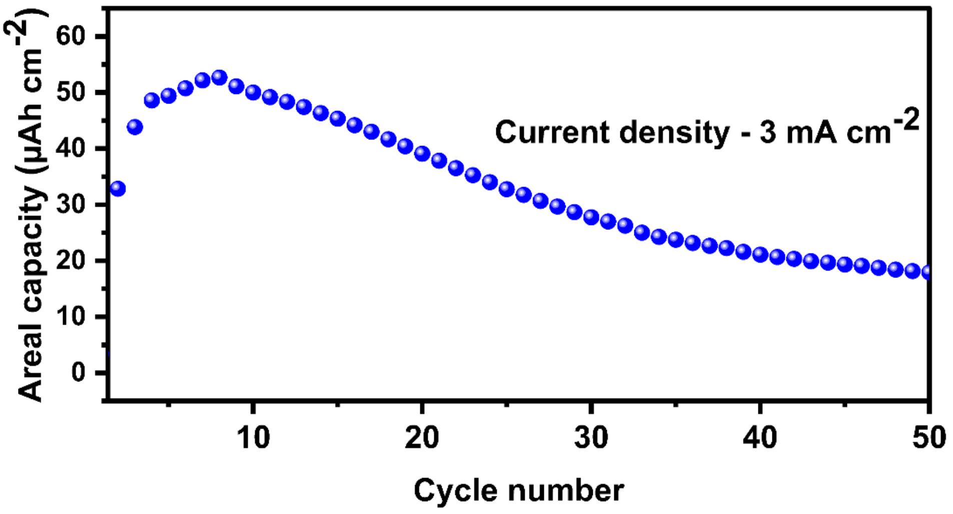

Figure S20. The GCD carried out for Pt/C at 3 mA cm^{-2} shows areal capacity >40 µAh cm^{-2} for around 20 cycles and >20 µAh cm^{-2} for 50 cycles.

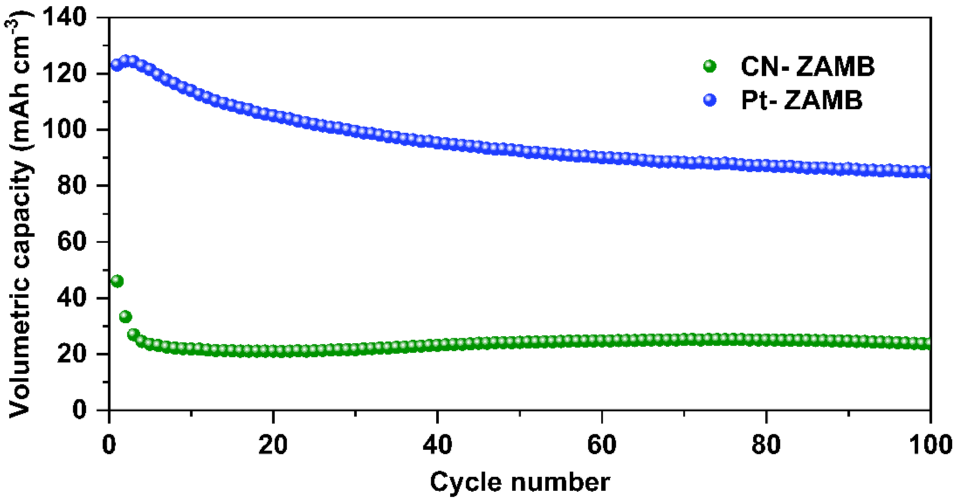

Figure S21. Rate test of the CN-ZAMB and Pt-ZAMB at areal current of 2000 µA cm^{-2} showing corresponding volumetric capacity that remains >85 mAh cm^{-3} for Pt-ZAMB and >25 mAh cm^{-3} for CN-ZAMB for 100 cycles.

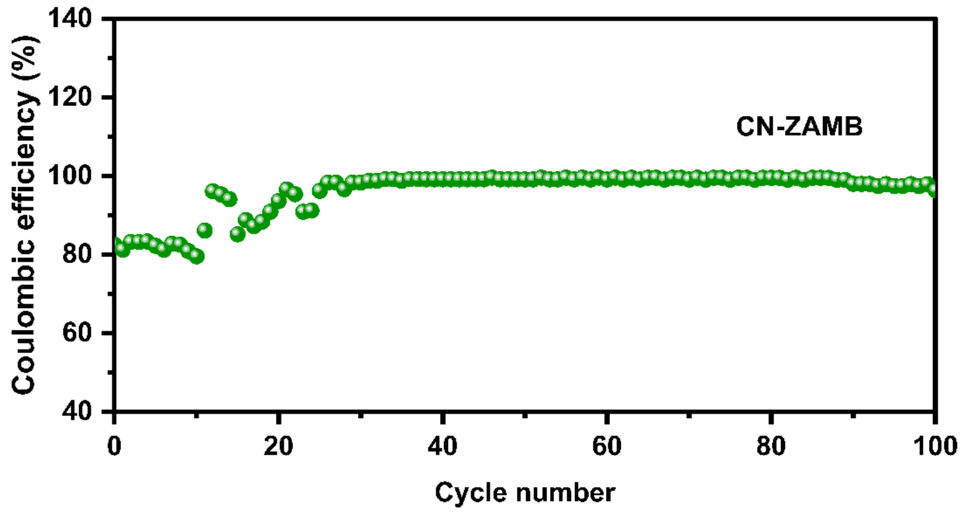

Figure S22. Coulombic efficiency data for CNZAMB shows an initial decrement then coming back to ~100% after 30 cycles.

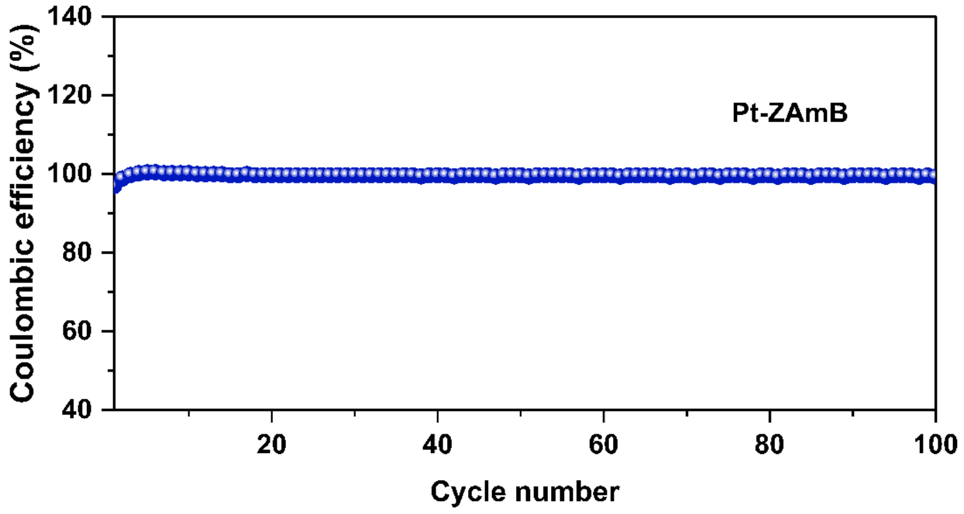

Figure S23. Coulombic efficiency data for CNZAMB shows ~100% retention for 100 cycles that shows no additional loss of current in any parasitic processes.

References

[1] S. Ghosh, S. S. Acharyya, R. Tiwari, B. Sarkar, R. K. Singha, C. Pendem, T. Sasaki, R. Bal, *ACS Catal* **2014**, *4*, 2169.

[2] M. A. Hamid, Y. Zengin, I. Boz, *Catal Commun* **2024**, *187*, DOI 10.1016/j.catcom.2024.106841.

[3] A. Singh, R. Sharma, A. Halder, *Nanoscale* **2024**, *16*, 4157.

[4] J. Zhou, W. Ma, Y. Mei, F. Wu, C. Xie, K. Wang, L. Zheng, L. Li, R. Chen, *Small Methods* **2024**, DOI 10.1002/smtd.202301411.

[5] Z. Wang, J. Huang, Z. Guo, X. Dong, Y. Liu, Y. Wang, Y. Xia, *Joule* **2019**, *3*, 1289.

[6] M. K. Okla, G. Harini, T. M. Dawoud, C. Akshhayya, A. Mohebaldin, A. A. AL-ghamdi, W. Soufan, M. A. Abdel-Maksoud, H. AbdElgawad, L. L. Raju, A. M. Thomas, S. Sudheer Khan, *Colloids Surf A Physicochem Eng Asp* **2022**, *641*.

[7] Mao, S.; Wen, Z.; Huang, T.; Hou, Y.; Chen, J. High-Performance Bi-Functional Electrocatalysts of 3D Crumpled Graphene–Cobalt Oxide Nanohybrids for Oxygen Reduction and Evolution Reactions. *Energy Environ. Sci.* **2014**, *7* (2), 609–616.

[8] Vineesh, T. V.; Kumar, M. P.; Takahashi, C.; Kalita, G.; Alwarappan, S.; Pattanayak, D. K.; Narayanan, T. N. Bifunctional Electrocatalytic Activity of Boron-Doped Graphene Derived from Boron Carbide. *Adv. Energy Mater.* **2015**, *5* (17), 1500658.
